# Health literacy of patients hospitalized for digestive surgeries*

**DOI:** 10.1590/1980-220X-REEUSP-2025-0267en

**Published:** 2026-03-16

**Authors:** Stéfani Rodrigues Venturini, Etiane de Oliveira Freitas, Silviamar Camponogara, Nara Marilene Oliveira Girardon-Perlini, Rosângela Marion da Silva, Liana Amorim Côrrea Trotte, Larissa Venturini

**Affiliations:** 1 Universidade Federal de Santa Maria, Departamento de Enfermagem, Santa Maria, RS, Brazil.; 2 Universidade Federal do Rio de Janeiro, Departamento de Enfermagem, Rio de Janeiro, RJ, Brazil.; 3 Universidade Federal de Santa Maria, Hospital Universitário de Santa Maria, Santa Maria, RS, Brazil.

**Keywords:** Health Literacy, Inpatients, Surgical Procedures, Operative, Digestive System, Perioperative Nursing

## Abstract

**Objectives:**

To assess the health literacy (HL) of patients in the pre- or postoperative period of digestive surgeries and to verify associations with sociodemographic and clinical variables.

**Method:**

A cross-sectional study was carried out at a public hospital. Data were collected from October 2023 to July 2024, through the application of a form with sociodemographic and clinical data and the Health Literacy Questionnaire (HLQ). Descriptive analyses, inferential statistical tests, and multiple linear regression were performed.

**Results:**

Of the 319 participants, 78.4% were in the postoperative period at the time of data collection. Among HLQ scales, “Ability to actively engage with healthcare providers” received the best score (3.70 ± 0.69), while “Having sufficient information to manage my health” (2.73 ± 0.29) had the worst score. Education level, race, age, having children, living with other people, and having a caregiver were associated with HL (p < 0.05). In adjusted analysis, the variables remained associated, except for race, income, and operative period.

**Conclusion:**

Interaction with professionals contributes to HL, while understanding and critically assessing health information weakens it. Lower education was the main factor associated with lower HL.

## INTRODUCTION

Health literacy (HL) emerges as a critical social determinant recognized by the World Health Organization as a central element for chronic non-communicable disease prevention, control, and management throughout the life course^(1)^. The most recent definition of the term HL was presented by the Health People 2030 initiative in the United States, which describes it as the individuals’ ability to find, understand, and use information and services to make decisions and take actions related to their own health and the health of others^(2)^. When insufficient, it is associated with lower adherence to therapeutic recommendations, difficulty in interpreting prescriptions, inappropriate use of medications, and barriers to the development of self-care practices, negatively impacting clinical outcomes and patient safety^(1,3)^. These aspects become especially relevant in the surgical context, where patients need to understand complex guidelines related to preoperative preparation, self-care in the postoperative period, and complications prevention.

Therefore, HL assessment is configured as an essential strategy in addressing health inequalities and improving care processes, especially in contexts that require a high degree of patient engagement^(2,3)^, such as the pre- and post-operative period of digestive surgeries. Digestive surgeries, frequently indicated in the management of complex conditions of the gastrointestinal tract, such as neoplasms, gastroesophageal reflux disease, cholelithiasis, pancreatitis, abdominal hernias, and intestinal syndromes, demand from patients an understanding of clinical aspects and behavioral and psychosocial adaptations^(4,5)^. The postoperative period, in particular, imposes substantial lifestyle changes, such as dietary modifications, adjustments to routine, management of devices such as stomas, catheters and drains, in addition to coping with the physical and emotional impacts resulting from the surgical intervention^(6,7)^.

International literature indicates that HL is a frequent problem among surgical patients. A meta-analysis of 40 studies, totaling 18,895 individuals, estimated that approximately one- third of this population presents inadequate HL (31.7%)^(8)^. Converging evidence indicates that this deficit is associated with unfavorable clinical outcomes, such as lower adherence to pre- and post-operative guidelines, longer hospital stays, increased readmission rates, and higher hospital costs^(9)^. Supporting these findings, a recent retrospective study of patients undergoing elective colorectal surgery identified that limited HL doubled the risk of postoperative complications, increased the incidence of surgical site infection, and prolonged hospital stay^(10)^. Although the review by Roy *et al*.^(8)^ consolidated evidence on HL in surgical populations, the authors highlight the scarcity of investigations conducted in Latin American countries, including Brazil, which limits the understanding of the local reality.

Despite global recognition of HL as a critical determinant of surgical safety, substantial gaps persist in Brazil, with no applied investigations in surgical patients identified in international reviews^(8)^. National research in perioperative nursing addresses aspects such as postoperative complications^(4)^, nutritional evolution^(5)^ and adaptation to surgical devices^(7)^, but has not yet directly explored the dimension of HL in this group.

Thus, this study adds unprecedented evidence by quantifying HL of hospitalized patients in the pre- or post-operative period of digestive surgeries in Brazil, contributing to the development of evidence-based care strategies, as well as enhancing the quality and equity of healthcare services. Given this context, this study aimed to assess HL of patients in the pre- or post-operative period of digestive surgeries and to verify associations with sociodemographic and clinical variables.

## METHOD

### Study Design

This is a cross-sectional study, designed according to STrengthening the Reporting of OBservational studies in Epidemiology guidelines.

### Setting

This is a public teaching hospital located in the central region of Rio Grande do Sul, serving as a regional reference center for medium and high complexity care within the Brazilian Health System, assisting approximately two million inhabitants in 45 municipalities. It has 380 active beds, more than 40 medical specialties, and high-complexity services. As a teaching hospital, it integrates care, teaching, research, and outreach. It stands out in the performance of surgical procedures, having performed 7,881 surgeries in 2019, approximately 800 of which were digestive tract surgeries^(11)^.

### Population and Selection Criteria

Participants were patients in the pre- or post-operative period of elective or emergency digestive surgery, hospitalized in the Surgical Clinic, Medical Clinic, and Adult Emergency Room units. The sample size was estimated using the Cochran method with correction for finite population, considering a population of 1,615 (patients undergoing digestive surgery in the two years prior to October 2023), a 95% confidence level (Z = 1.96), a 5% margin of error, and a 50% expected proportion, obtaining a minimum sample size of 311 participants. The selected sample was intentional and based on convenience. Daily, members of the research team tracked patients who met the inclusion/exclusion criteria in the local study units using information from electronic medical records, creating a list of potential participants who were approached sequentially.

Patients aged 18 years or older, hospitalized in the aforementioned units, in the pre- or post-operative period of digestive surgeries, were included. Those who presented limitations in communication and interaction, such as difficulty speaking, excessive sleepiness, or significant emotional instability, were excluded. The final sample consisted of 319 individuals.

### Data Collection

Data collection was conducted daily from October 2023 to July 2024 by a principal investigator and research assistants (graduate students) who received prior theoretical and practical training. Before beginning data collection, the study was presented individually, and any questions were answered by potential participants. Informed Consent Forms were obtained from those who agreed to participate in the research. In a private setting, ensuring confidentiality, the interviewer orally administered the sociodemographic and clinical characterization form and the Health Literacy Questionnaire – Brazilian version (HLQ-Br). It is important to note that, as mentioned in the selection criteria, participants answered the survey regardless of the operative period (pre- or post-), and this was also a variable analyzed.

HLQ was developed by Australian authors, translated and adapted into Brazilian Portuguese, resulting in the HLQ-Br version^(12)^. HLQ-Br demonstrated good psychometric properties in different population contexts. Validity studies showed adequate internal reliability, with satisfactory Cronbach’s alpha coefficients (greater than 0.70), as well as a factorial structure consistent with the original model^(12)^. For its use in this study, formal authorization was requested from the Australian university to use the Brazilian version of the instrument (e-mail: hl-info@swin.edu.au).

It is a multidimensional instrument, containing 44 items arranged in nine scales, which can be self-administered or applied through interviews. It is divided into two parts: the first part (scales 1 to 5) assesses the perception of skills and resources to find, understand, assess, and use health information; and the responses in this part range from “strongly disagree” (1) to “strongly agree” (4). The second part (scales 6 to 9) assesses the ease of access to healthcare services and care, and the responses range from “always difficult” (1) to “always easy” (5)^(13)^. To facilitate participants’ understanding of the response options, an illustrative ruler produced by the researcher was used when necessary.

The scales assessed are: scale 1 – Feeling understood and supported by healthcare providers (four items); scale 2 – Having sufficient information to manage my health (four items); scale 3 – Actively managing my health (five items); scale 4 – Social support for health (five items); scale 5 – Appraisal of health information (five items); scale 6 – Ability to actively engage with healthcare providers (five items); scale 7 – Navigating the healthcare system (six items); scale 8 – Ability to find good health information (five items); scale 9 – Understand health information enough to know what to do (five items)^(12)^.

### Study Variables

Dependent variables corresponded to the nine scales of HLQ-Br, treated as continuous mean scores (part 1: 1–4; part 2: 1–5). Independent variables included sociodemographic aspects (age, sex, race/color, marital status, education, family income, having children, number of residents in the household) and clinical/care aspects (presence of a companion during hospitalization, comorbidities, use of assistive devices, perioperative period: pre- or postoperative, length of stay, and type of surgery).

### Data Analysis

Data were analyzed using the Statistical Package for the Social Sciences version 22. Descriptive statistics were used for sociodemographic and clinical characterization. HLQ-Br does not provide a global assessment for the questionnaire. Each scale was scored by the mean of its items, with the highest mean indicating potential and the lowest indicating fragility of HL^(12)^. Internal consistency was measured using Cronbach’s alpha, which showed it to be adequate (α = 0.82 in part 1 and 0.85 in part 2).

For bivariate analysis, the Mann-Whitney test and the Kruskal-Wallis test were applied, followed by Dunn’s multiple comparisons, since the Shapiro-Wilk normality test indicated data with an abnormal distribution (p < 0.05). Subsequently, the independent variables that showed a statistically significant association with HLQ scales (p < 0.05) were selected for multiple linear regression modeling. Thus, nine multiple linear regressions were performed, following the assumptions of this type of analysis (independence of residuals, absence of multicollinearity, absence of outliers, normally distributed residuals, and homoscedasticity).

### Ethical Aspects

The study was approved by the *Universidade Federal de Santa Maria* Research Ethics Committee, under Opinion 6,423,890, and conducted in accordance with the ethical guidelines established by Resolutions 466/2012 and 510/2016 of the Brazilian National Health Council. All participants signed the Informed Consent Form.

## RESULTS

The study included 319 patients, with a mean age of 53.25 (SD = 17.43) years. The majority were male (53.9%), self-identified as white (73.7%), had a partner (68.7%), and had children (68.7%). Concerning socioeconomic aspects, 51.4% had a family income of up to three minimum wages, and 59.6% had education up to elementary school. The majority were accompanied during hospitalization (90.3%).

Regarding clinical conditions, 65.5% reported having some comorbidity, and 78.4% were in the postoperative period at the time of data collection. As for the surgical procedures performed by participants, laparotomy (n = 74; 23.2%), appendectomy (n = 64; 20.1%), cholecystectomy (n = 56; 17.6%), and colectomy (n = 15; 4.7%) prevailed.

Through descriptive analysis of HQL-Br, the following were identified as strengths of HL: “Social support for health”, with a mean of 3.25 ± 0.44, in part 1, and “Ability to actively engage with healthcare providers”, with a mean of 3.70 ± 0.69, in part 2. Meanwhile, the limitations found were “Having sufficient information to manage my health”, with a mean of 2.73 ± 0.29, in part 1, and “Ability to find good health information”, with a mean of 3.39 ± 0.71, in part 2, as described in Table 1.

**Table 1:** Descriptive measures of the Health Literacy Questionnaire – Brazilian version of 319 patients hospitalized for digestive surgery – Santa Maria, RS, Brazil, 2024.

HLQ-Br scales	Number of items	Mean (SD)^1^	95%CI
**Part 1**			
1 – Feeling understood and supported by healthcare providers	4	2.94 (0.53)	(2.88–3.00)
2 – Having sufficient information to manage my health	5	2.73 (0.29)	(2.67–2.79)
3 – Actively managing my health	5	2.76 (0.44)	(2.71–2.81)
4 – Social support for health	5	3.25 (0.44)	(3.20–3.30)
5 – Appraisal of health information	5	2.82 (0.46)	(2.77–2.87)
**Part 2**			
6 – Ability to actively engage with healthcare providers	5	3.70 (0.69)	(3.63–3.78)
7 – Navigating the healthcare system	6	3.42 (0.65)	(3.35–3.49)
8 – Ability to find good health information	5	3.39 (0.71)	(3.31–3.47)
9 – Understand health information enough to know what to do	5	3.58 (0.69)	(3.50–3.66)

Note: data presented as mean (SD – standard deviation). Additional values in parentheses in the corresponding column indicate the 95% Confidence Interval (95%CI).

Source: research data (2024).

Tables 2 and 3 present the results of the bivariate analysis between HLQ-Br scales and participant sociodemographic and clinical variables, corresponding to part 1 and part 2 of the instrument, respectively. In part 1, it was identified that non-white people, with low education levels and without comorbidities reported less understanding and support from healthcare providers. Women, those with only elementary school, and those without pre-existing conditions also felt less informed about how to take care of their own health.

**Table 2:** Bivariate analysis of part 1 of the Health Literacy Questionnaire – Brazilian version with sociodemographic and clinical variables – Santa Maria, RS, Brazil, 2024.

Variables	HLQ-Br scales Part 1 (scores 1–4)				
	Scale 1	Scale 2	Scale 3	Scale 4	Scale 5
**Sex** ^+^					
Female	2.91 (0.54)	2.67 (0.56)	2.74 (0.46)	3.20 (0.45)	2.80 (0.43)
Male	2.97 (0.54)	2.78 (0.52)	2.78 (0.43)	3.30 (0.44)	2.84 (0.48)
P-value	0.248	**0.029**	0.442	**0.042**	0.462
**Race**					
White	2.98 (0.56)	2.73 (0.55)	2.79 (0.44)	3.28 (0.44)	2.84 (0.45)
Non-white	2.83 (0.45)	2.74 (0.52)	2.66 (0.45)	3.16 (0.45)	2.76 (0.49)
P-value	**0.003**	0.671	**0.031**	**0.020**	0.191
**Marital status**					
With companion	2.95 (0.49)	2.71 (0.52)	2.77 (0.43)	3.28 (0.41)	2.80 (0.44)
Without a partner	2.90 (0.61)	2.76 (0.58)	2.71 (0.45)	3.18 (0.50)	2.84 (0.49)
P-value	0.799	0.398	0.163	0.088	0.601
**Education** ^#^					
Illiterate or elementary school	2.87 (0.51)^b^	2.64 (0.52)^b^	2.78 (0.42)	3.24 (0.41)^b^	2.70 (0.44)^c^
High school	2.97 (0.56)^b^	2.83 (0.52)^a^	2.68 (0.47)	3.19 (0.51)^b^	2.93 (0.40)^b^
Higher education or graduate studies	3.24 (0.50)^a^	2.95 (0.57)^a^	2.83 (0.44)	3.48 (0.34)^a^	3.19 (0.41)^a^
P-value	**0.002**	**0.004**	0.066	**0.004**	**< 0.001**
**Has children**					
Yes	2.94 (0.53)	2.71 (0.55)	2.75 (0.46)	3.26 (0.44)	2.80 (0.46)
No	2.95 (0.58)	2.83 (0.48)	2.79 (0.38)	3.23 (0.49)	2.91 (0.44)
P-value	0.519	0.160	0.708	0.894	0.143
**Income** ^#^					
Up to one MW	2.84 (0.54)	2.67 (0.56)	2.76 (0.45)	3.14 (0.49)^b^	2.78 (0.47)
Two or three MWs	2.95 (0.55)	2.74 (0.54)	2.78 (0.44)	3.27 (0.43)^a^	2.80 (0.45)
> three MWs	3.03 (0.48)	2.78 (0.52)	2.73 (0.44)	3.33 (0.42)^a^	2.92 (0.47)
P-value	0.333	0.595	0.577	**0.034**	0.079
**Has a companion at the hospital**					
Yes	2.96 (0.51)	2.73 (0.53)	2.74 (0.45)	3.27 (0.42)	2.81 (0.46)
No	2.77 (0.68)	2.68 (0.58)	2.90 (0.39)	3.00 (0.58)	2.87 (0.47)
P-value	0.329	0.997	0.094	**0.010**	0.683
**Has comorbidities**					
Yes	3.02 (0.48)	2.77 (0.52)	2.80 (0.44)	3.27 (0.44)	2.82 (0.46)
No	2.79 (0.60)	2.64 (0.56)	2.68 (0.44)	3.22 (0.45)	2.81 (0.47)
P-value	**< 0.001**	**0.017**	**0.011**	0.213	0.661
**Current period**					
Preoperative	2.97 (0.58)	2.72 (0.53)	2.86 (0.36)	3.21 (0.44)	2.83 (0.48)
Postoperative	2.93 (0.52)	2.73 (0.54)	2.73 (0.64)	3.26 (0.45)	2.82 (0.45)
P-value	0.323	0.822	**0.028**	0.273	0.904

Note: scale 1 – Feeling understood and supported by healthcare providers; scale 2 – Having sufficient information to manage my health; scale 3 – Actively managing my health; scale 4 – Social support for health; scale 5 – Appraisal of health information; MW – minimum wage; +Mann-Whitney U test; #Kruskal-Wallis test; data presented as mean (standard deviation – SD). Additional values in parentheses in the corresponding column indicate the 95% Confidence Interval (95%CI). Superscript letters (a, b, c) indicate significant differences between groups according to Dunn’s multiple comparisons (p < 0.05); groups that do not share the same letter differ significantly.

Source: research data (2024).

**Table 3:** Bivariate analysis of part 2 of the Health Literacy Questionnaire – Brazilian version with sociodemographic and clinical variables – Santa Maria, RS, Brazil, 2024.

Variables	HLQ-Br scales, part 2 (scores 1–4)			
	Scale 6	Scale 7	Scale 8	Scale 9
**Sex** ^+^				
Female	3.68 (0.71)	3.37 (0.73)	3.38 (0.78)	3.63 (0.69)
Male	3.73 (0.68)	3.46 (0.57)	3.40 (0.65)	3.54 (0.70)
P-value	0.484	0.209	0.900	0.132
**Race**				
White	3.72 (0.67)	3.40 (0.67)	3.41 (0.68)	3.58 (0.63)
Non-white	3.66 (0.76)	3.49 (0.59)	3.33 (0.79)	3.59 (0.85)
P-value	0.739	0.254	0.444	0.825
**Marital status**				
With a partner	3.66 (0.68)	3.40 (0.61)	3.37 (0.67)	3.56 (0.70)
Without a partner	3.78 (0.70)	3.45 (0.72)	3.42 (0.78)	3.62 (0.67)
P-value	0.085	0.397	0.354	0.495
**Education** ^#^				
Illiterate or elementary school	3.65 (0.67)	3.34 (0.61)b	3.17 (0.68)c	3.41 (0.72)b
High school	3.72 (0.74)	3.49 (0.69)ab	3.64 (0.63)b	3.78 (0.58)a
Higher education or graduate studies	3.93 (0.67)	3.66 (0.67)a	3.94 (0.48)a	3.96 (0.51)a
P-value	0.078	**0.011**	**< 0.001**	< 0.001
**Has children**				
Yes	3.67 (0.71)	3.38 (0.66)	3.33 (0.70)	3.51 (0.69)
No	3.88 (0.62)	3.59 (0.58)	3.67 (0.71)	3.93 (0.61)
P-value	**0.041**	**0.032**	**0.001**	**< 0.001**
**Income** ^#^				
Up to one MW	3.67 (0.68)	3.42 (0.72)	3.26 (0.75)b	3.41 (0.78)
Two or three MWs	3.69 (0.72)	3.40 (0.64)	3.36 (0.72)b	3.62 (0.70)
> three MWs	3.77 (0.65)	3.46 (0.61)	3.61 (0.57)a	3.69 (0.54)
P-value	0.730	0.793	**0.005**	0.061
**Has a companion at the hospital**				
Yes	3.69 (0.70)	3.42 (0.66)	3.37 (0.70)	3.56 (0.70)
No	3.83 (0.60)	3.43 (0.60)	3.57 (0.78)	3.80 (0.62)
P-value	0.337	0.905	0.189	0.109
**Has comorbidities**				
Yes	3.67 (0.68)	3.44 (0.66)	3.31 (0.70)	3.48 (0.67)
No pathology	3.76 (0.73)	3.39 (0.63)	3.55 (0.69)	3.77 (0.70)
P-value	0.178	0.276	**0.004**	**0.001**
**Current period**				
Preoperative	3.86 (0.64)	3.47 (0.66)	3.44 (0.77)	3.55 (0.64)
Postoperative	3.66 (0.70)	3.40 (0.65)	3.38 (0.69)	3.59 (0.71)
P-value	**0.044**	0.345	0.521	0.441

Note: scale 6 – Ability to actively engage with healthcare providers; scale 7 – Navigating the healthcare system; scale 8 – Ability to find good health information; scale 9 – Understand health information enough to know what to do; MW – minimum wage; +Mann-Whitney U test; #Kruskal-Wallis test; data presented as mean (standard deviation – SD). Additional values in parentheses in the corresponding column indicate the 95% Confidence Interval (95%CI). Superscript letters (a, b, c) indicate significant differences between groups according to Dunn’s multiple comparisons (p < 0.05); groups that do not share the same letter differ significantly.

Source: research data (2024).

As for “Actively managing my health”, frailty was more evident in non-white patients, those without comorbidities, and those who were in the postoperative period at the time of data collection. “Social support for health” was lower among women, non-white individuals, those with an income of up to one minimum wage, and hospitalized patients without a companion. “Ability to find good health information” was lower in participants with lower educational levels and those using assistive devices.

In part 2 of HLQ-Br, it was evident that those with children exhibited worse scores on all scales. Data on educational level showed that participants with elementary school were the most vulnerable in “Navigating the healthcare system”, “Ability to find good health information”, and “Understand health information enough to know what to do”. Family income of up to three minimum wages was associated with greater difficulty in locating good information, and participants with comorbidities showed less ability to find and understand health information. Furthermore, those in the postoperative period reported a lower capacity for active interaction with professionals.

Table 4 shows the correlations between HLQ and the quantitative variables investigated, such as age, length of stay, and number of people living in the residence. A weak positive correlation is observed between age and scales 1, 2, 3, and 4; a weak negative correlation between scales 5 and 8; and a moderate correlation with scale 9. Furthermore, a weak negative correlation is observed between length of stay and scales 3, 6, 8, and 9, and also a weak negative correlation between the number of residents in the household and scales 2, 3, and 6.

**Table 4:** Correlation between the scales of the Health Literacy Questionnaire – Brazilian version with quantitative variables – Santa Maria, RS, Brazil, 2024.

HLQ-Br dimensions	Age (years)		Length of hospital stay (days)		Number of people living in the residence	
	r	p-value	r	p-value	r	p-value
**Part 1 (scores 1–4)**						
1 – Feeling understood and supported by healthcare providers	0.207**	**< 0.001**	-0.067	0.234	-0.091	0.104
2 – Having sufficient information to manage my health	0.117*	**0.037**	-0.082	0.147	−0.153*	**0.006**
3 – Actively managing my health	0.167**	**0.003**	−0.119*	**0.035**	−0.200*	**< 0.001**
4 – Social support for health	0.144*	**0.01**	0.031	0.577	-0.005	0.927
5 – Appraisal of health information	−0.124*	**0.027**	-0.1	0.076	-0.039	0.489
**Part 2 (scores 1–5)**						
6 – Ability to actively engage with healthcare providers	0.022	0.696	−0.130*	**0.021**	−0.116*	**0.038**
7 – Navigating the healthcare system	0.099	0.077	-0.062	0.27	-0.065	0.244
8 – Ability to find good health information	−0.238**	**< 0.001**	−0.152*	**0.007**	0.033	0.562
9 – Understand health information enough to know what to do	−0.332**	**< 0.001**	−0.153*	**0.007**	0.066	0.239

r: Spearman correlation coefficient *p < 0.05 and **p < 0.001.

Source: research data (2024).

After adjustment using multiple linear regression, education level and age were significantly associated with “Feeling understood and supported by healthcare providers”. Participants with educational levels below higher education had lower scores on this dimension, including those who were illiterate (coeffb = -0.87; 95%CI: -1.41 to -0.33), those with elementary school (coeffb = -0.49; 95%CI: -0.69 to -0.30), and those with high school (coeffb = -0.28; 95%CI: -0.48 to -0.08). Older participants tended to perceive better under­standing and greater professional support (coeffb = 0.008; 95%CI: -0.004 to 0.012).

On “Having sufficient information to manage my health”, education level and number of people in the household were associated. Illiterate participants (coeffb = -0.56; 95%CI: -1.11 to -0.01) and those with elementary school (coeffb = -0.40; 95%CI: -0.60 to -0.20) showed a lower perception of access to information. Furthermore, the number of people living in the household was inversely associated with this perception (coeffb = -0.051; 95%CI: -0.100 to -0.001). Regarding “Actively managing my health”, the number of people living in the household was the only associated factor (coeffb = -0.060; 95%CI: -0.101 to -0.018), indicating that living with more people was related to less active engagement in one’s own care.

For “Social support for health”, sex, education level, presence of a caregiver, and age were associated variables. Women presented lower social support (coeffb = -0.10; 95%CI: -0.20 to -0.01), as did participants with elementary (coeffb = -0.29; 95%CI: -0.47 to -0.11) and high (coeffb = -0.31; 95%CI: -0.49 to -0.13) school, compared to those with higher education. Having a caregiver was associated with greater support (coeffb = 0.22; 95%CI: 0.06 to 0.38), and age showed a positive relationship with this dimension (coeffb = 0.004; 95%CI: 0.001 to 0.007). Education level was the only factor associated with “Appraisal of health information”, with lower scores among illiterate individuals (coeffb = -1.00; 95%CI: -1.45 to -0.54), participants with elementary school (coeffb = -0.49; 95%CI: -0.65 to -0.32) and high school (coeffb = -0.26; 95%CI: -0.43 to -0.09).

Regarding part 2 of the instrument, “Ability to actively engage with healthcare providers” was associated only with the “having children” variable (coeffb = -0.21; 95%CI: -0.42 to -0.01). Education level also influenced “Navigating the healthcare system”, with lower scores among those with elementary school (coeffb = -0.29; 95%CI: -0.53 to -0.04). Finally, on “Ability to find good health information”, participants who were illiterate (coeffb = -0.96; 95%CI: -1.68 to -0.24) and those with elementary school (coeffb = -0.64; 95%CI: -0.91 to -0.37) also presented lower scores. On “Understand health information enough to know what to do”, lower scores were observed among illiterate individuals (coeffb = -1.29; 95%CI: -1.97 to -0.61) and participants with elementary school (coeffb = -0.38; 95%CI: -0.63 to -0.14), in addition to an inverse association with age (coeffb = -0.004; 95%CI: -0.011 to -0.001).

## DISCUSSION

This study identified, as strengths of HL, interaction with professionals and social support, and as weaknesses, insufficient information for self-care and difficulty in finding quality information. Multivariate analysis showed that education level was the main factor associated with the different domains of HLQ-Br, reinforcing the influence of the educational gradient on HL. Variables such as age, presence of children, number of residents in the household, and comorbidities also showed relevant associations, revealing important nuances for understanding surgical patients’ vulnerability.

The multidimensional structure of HLQ-Br allows for a comprehensive assessment that transcends the analysis of mere reading and text interpretation skills, incorporating essential aspects such as navigation within the healthcare system, interaction with professionals, and perception of social support. This characteristic makes it possible to accurately identify areas of competence already consolidated, such as the adequate understanding of health information, the ability to locate reliable sources, effective interaction with professionals, organized navigation through services, and the use of support networks, and, simultaneously, weaknesses that demand the targeting of interventions sensitive to patients’ socioeconomic and cultural particularities^(14)^.

Analysis of the scales highlighted HL’s potential, i.e., aspects that are strengthened, such as “Social support for health” and “Ability to actively engage with healthcare providers”, indicating that support networks, both family and institutional, can play a protective role in the face of the challenges posed by hospitalization. Robust evidence indicates that individuals who perceive themselves as supported tend to have better therapeutic adherence, greater engagement in self-care, and a reduced risk of complications^(15,16)^, reaffirming the positive impact of social support on health outcomes.

The greatest limitations of HL identified in the patients of this study were “Having sufficient information to manage my health” and “Ability to find good health information”, indicating significant difficulties in accessing and using information for self-care. This scenario is especially critical in the context of digestive surgeries, which require complex lifestyle changes and rigorous monitoring for complications. Therefore, this may be another contributing factor to increased information scarcity in the postoperative period, hindering the identification of care failures. Traditional clinical measures do not fully capture the patient experience, limiting person-centered quality assessment^(17)^.

Multivariate analysis confirmed that education level was the variable most strongly associated with the HLQ-Br dimensions, even after adjusting for other factors, supporting evidence that low education level is an important predictor of lower levels of HL^(18,19)^. Patients with only elementary school consistently presented the worst scores across several dimensions, followed by those with high school, while those with higher education or graduate degrees demonstrated better performance. This educational gradient highlights the relationship between HL and social inequities, as it limits access to, understanding of, and use of health information^(20)^. These findings are consistent with previous studies, which describe how educational deficits can negatively impact aspects such as autonomy and informed decision-making, although these outcomes were not directly assessed in this study^(18,19)^.

Sociodemographic analysis added important, though not conclusive, elements. Although the literature frequently relates advanced age to lower levels of HL, in this study, it was observed that older patients tended to present higher scores in dimensions linked to social support and support from healthcare providers, suggesting that contextual factors may attenuate or modify this pattern. This finding is supported by a study^(21)^ that highlights the role of social support as a positive mediator of the relationship between aging and engagement in self-care, mitigating, in part, the limitations associated with age.

Another aspect that deserves attention refers to the lower mean scores identified among patients who had children, especially on scales of interaction with professionals, navigation within the healthcare system, and comprehension of information, suggesting greater vulnerability in HL dimensions. This result may be related to the overload of family responsibilities, which frequently falls on these individuals and can reduce the time, attention, and cognitive resources dedicated to their own health needs. This interpretation is supported by the literature, which associates the accumulation of family roles and, in particular, gender inequalities with a lower capacity for engagement in self-care practices and the search for health information^(22,23)^.

Particularly relevant was the finding that postoperative patients presented marked frailty in HL with regard to “Actively managing my health” and “Ability to actively engage with healthcare providers”, a finding consistent with international literature^(15,16)^. This finding reinforces the need for the perioperative nursing team to systematically incorporate HL assessment into care planning in order to prevent communication failures and support adherence to clinical recommendations.

“Ability to find good health information”, which presented one of the lowest means in this study, deserves special attention, especially given the contemporary context of widespread circulation of information that is often inaccurate, incomplete, or incorrect. This scenario exposes patients with low HL to the risk of misinformation, adoption of unsafe practices, inappropriate use of therapies, and a higher probability of adverse outcomes^(24,25)^.

Given this scenario, it becomes evident that healthcare services need to adopt systematic, continuous, and structured strategies to assess and strengthen surgical patients’ HL. To ensure safety, autonomy, and adherence to care, it is essential to adopt practices such as clear communication, the use of visual educational materials, and validity of comprehension through methodologies such as teach-back, in addition to longitudinal educational support^(26,27)^.

Within this framework, in order to educate patients, the nurse’s role in the context of surgical patients can benefit, above all, from the teach-back method, a communication strategy that promotes understanding, adherence, and safety in care^(28)^. In the pre-operative phase, teach-back allows verification of whether the patient has correctly understood instructions regarding fasting, hygiene, medication use, and emotional preparation for surgery. In the post-operative phase, this technique assists in explaining wound care, medication administration, recognizing signs of complications, and providing guidance for returning to daily activities. By asking patients to repeat the information in their own words, nurses confirm understanding and identifies potential doubts, promoting safety, autonomy, and adherence to treatment, as well as contributing to a faster and more effective recovery.

Additionally, the findings reinforce the strategic role of the nursing team, which, due to its proximity to patients, occupies a privileged position in identifying barriers to HL and in developing educational interventions, directly contributing to the improvement of care and the reduction of postoperative risks^(29)^.

Finally, it is worth highlighting that, despite the growing international appreciation of HL as a central component of care practices, there are still gaps in knowledge production on the subject in the Brazilian context, especially in surgical populations, which limits the development of evidence-based policies and practices.

This study presents some limitations, one of which is related to its cross-sectional design, which does not allow for establishing causal relationships, restricting itself to identifying associations. The convenience sample from a single teaching hospital also limits the representativeness and generalizability of the findings. Although HLQ-Br is a validated instrument in Brazil, it lacks established cut-off points that allow for the categorization of the HL level, restricting the interpretation of results to mean scores. Furthermore, the use of visual aids to support the understanding of items represented a methodological adaptation that may have influenced the responses. The possibility of selection bias, due to the non-recruitment of patients with communication limitations, and information bias, since part of the data was self-reported, should also be highlighted. Despite controlling for confounding through multiple regression, the influence of unmeasured variables cannot be ruled out.

Despite its limitations, this study makes relevant contributions. In patient care, it supports the perioperative nursing team in identifying patients who are more vulnerable in relation to HL and in planning individualized educational interventions, using clear language, teach-back, and adapted materials. In management, it supports the incorporation of HL assessment as an indicator of hospital quality and safety, guiding institutional policies and training programs. In this research, it expands national evidence on HL in surgical patients, stimulating multicenter and longitudinal studies. Finally, it favors the development of personalized practices, tailored according to sociodemographic and clinical profiles, such as differentiated strategies for individuals with low levels of education, reinforcement of social support for patients without a companion, and greater postoperative follow-up for vulnerable groups.

## CONCLUSION

The study identified the interaction with professionals and social support as strengths of HL. Weaknesses identified included insufficient information for self-care and difficulty in finding quality information in the context of surgical patients in the pre- or post-operative period.

Multivariate analysis revealed that lower education level was the main factor associated with worse scores in several dimensions, while variables such as age, presence of children, number of residents in the household, comorbidities, and perioperative period also showed specific associations.

These findings demonstrate the influence of educational gradient and social conditions on HL, reinforcing the importance of incorporating its systematic assessment into perioperative nursing practice and developing accessible, patient-centered educational interventions.

## DATA AVAILABILITY

The entire dataset supporting the results of this study is available upon request to the corresponding author.
