# Supplementary material for: Health literacy of patients hospitalized for digestive surgeries
Source: Rev Esc Enferm USP. 2026 Mar 16;60:e20250267. doi: 10.1590/1980-220X-REEUSP-2025-0267en (PMC12991410; doi:10.1590/1980-220X-REEUSP-2025-0267en)
Supplement: Translation

# Translation: Letramento em saúde de pacientes hospitalizados por cirurgias digestivas*

**DOI:** 10.1590/1980-220X-REEUSP-2025-0267pt

**Authors:** Stéfani Rodrigues Venturini, Etiane de Oliveira Freitas, Silviamar Camponogara, Nara Marilene Oliveira Girardon-Perlini, Rosângela Marion da Silva, Liana Amorim Côrrea Trotte, Larissa Venturini

**Affiliations:** 1 Universidade Federal de Santa Maria, Departamento de Enfermagem, Santa Maria, RS, Brasil.; 2 Universidade Federal do Rio de Janeiro, Departamento de Enfermagem, Rio de Janeiro, RJ, Brasil.; 3 Universidade Federal de Santa Maria, Hospital Universitário de Santa Maria, Santa Maria, RS, Brasil.

**Keywords:** Literacia para a Saúde, Pacientes Internados, Procedimentos Cirúrgicos Operatórios, Sistema Digestório, Enfermagem Perioperatória

## Abstract

**Objetivos:**

Avaliar o letramento em saúde (LS) de pacientes no período pré- ou pós-operatório de cirurgias digestivas e verificar associações com variáveis sociodemográficas e clínicas.

**Método:**

Estudo transversal, realizado em hospital público. Os dados foram coletados no período de outubro de 2023 a julho de 2024, por meio da aplicação de formulário com dados sociodemográficos e clínicos e do *Health Literacy Questionnaire* (HLQ). Realizaram-se análises descritivas, testes estatísticos inferenciais e regressão linear múltipla.

**Resultados:**

Dos 319 participantes, 78,4% estavam no pós-operatório no momento da coleta. Entre as escalas do HLQ, “Capacidade de interagir ativamente com os profissionais de saúde” obteve melhor avaliação (3,70 ± 0,69), enquanto “Informações suficientes para cuidar da saúde” (2,73 ± 0,29) apresentou pior avaliação. Escolaridade, raça, idade, ter filhos, morar com outras pessoas e ter cuidador se associaram ao LS (p < 0,05). Na análise ajustada, as variáveis mantiveram associação, exceto raça, renda e período operatório.

**Conclusão:**

A interação com profissionais contribui para o LS, enquanto a compreensão e a avaliação crítica das informações em saúde fragilizam-no. A menor escolaridade foi o principal fator associado a um menor LS.

## INTRODUÇÃO

O letramento em saúde (LS) emerge como um determinante social crítico reconhecido pela Organização Mundial da Saúde como elemento central para a prevenção, controle e manejo das doenças crônicas não transmissíveis ao longo do curso da vida^(1)^. A definição mais recente do termo LS foi apresentada pela iniciativa *Health People 2030*, nos Estados Unidos, que o descreve como a capacidade que os indivíduos têm de encontrar, compreender e usar informações e serviços para tomar decisões e ações relacionadas à saúde para si mesmos e para os outros^(2)^. Quando insuficiente, associa-se à menor adesão às recomendações terapêuticas, dificuldade na interpretação de prescrições, uso inadequado de medicamentos e barreiras no desenvolvimento de práticas de autocuidado, impactando negativamente os desfechos clínicos e a segurança do paciente^(1,3)^. Esses aspectos ganham especial relevância no contexto cirúrgico, em que o paciente precisa compreender orientações complexas relacionadas ao preparo pré-operatório, ao autocuidado no período pós-operatório e à prevenção de complicações.

A avaliação do LS, portanto, configura-se como uma estratégia essencial no enfrentamento das desigualdades em saúde e na qualificação dos processos de cuidado, especialmente em contextos que exigem alto grau de engajamento do paciente^(2,3)^, como o período pré- e pós-operatório de cirurgias digestivas. As cirurgias digestivas, frequentemente indicadas no manejo de condições complexas do trato gastrointestinal, como neoplasias, doença do refluxo gastroesofágico, colelitíase, pancreatite, hérnias abdominais e síndromes intestinais, demandam dos pacientes a compreensão dos aspectos clínicos e adaptações comportamentais e psicossociais^(4,5)^. O pós-operatório, em especial, impõe mudanças substanciais no estilo de vida, como modificações na dieta, ajustes na rotina, manejo de dispositivos, como estomias, sondas e drenos, além do enfrentamento dos impactos físicos e emocionais decorrentes da intervenção cirúrgica^(6,7)^.

A literatura internacional aponta que a limitação do LS é um problema frequente entre pacientes cirúrgicos. Meta-análise de 40 estudos, totalizando 18.895 indivíduos, estimou que aproximadamente um terço dessa população apresenta LS inadequado (31,7%)^(8)^. Evidências convergentes indicam que esse déficit se associa a desfechos clínicos desfavoráveis, como menor adesão às orientações pré- e pós-operatórias, maior tempo de internação, incremento das taxas de readmissão e elevação dos custos hospitalares^(9)^. Corroborando esses achados, estudo retrospectivo recente com pacientes submetidos à cirurgia colorretal eletiva identificou que o LS limitado duplicou o risco de complicações pós-operatórias, aumentou a incidência de infecção de sítio cirúrgico e prolongou a permanência hospitalar^(10)^. Embora a revisão de Roy et al.^(8)^ tenha consolidado evidências sobre o LS em populações cirúrgicas, os autores destacam a escassez de investigações conduzidas em países da América Latina, incluindo o Brasil, o que limita a compreensão da realidade local.

Apesar do reconhecimento global do LS como determinante crítico da segurança cirúrgica, no Brasil, persistem lacunas substantivas, não se identificando investigações aplicadas em pacientes cirúrgicos em revisões internacionais^(8)^. Pesquisas nacionais em enfermagem perioperatória abordam aspectos como complicações pós-operatórias^(4)^, evolução nutricional^(5)^ e adaptação a dispositivos cirúrgicos^(7)^, mas ainda não exploraram diretamente a dimensão do LS nesse grupo.

Assim, este estudo acrescenta evidências inéditas ao quantificar o LS de pacientes hospitalizados no período pré- ou pós- operatório de cirurgias digestivas no Brasil, contribuindo para fundamentar estratégias de cuidado orientadas por evidências científicas, além de potencializar a qualidade e equidade dos serviços de saúde. Diante desse panorama, este estudo tem como objetivo avaliar o LS de pacientes no período pré- ou pós-operatório de cirurgias digestivas e verificar associações com variáveis sociodemográficas e clínicas.

## MÉTODO

### Desenho do Estudo

Trata-se de estudo transversal, delineado segundo as diretrizes do *STrengthening the Reporting of OBservational studies in Epidemiology*.

### Cenário

Trata-se de hospital público e de ensino, localizado na região central do Rio Grande do Sul, sendo referência regional em média e alta complexidade pelo Sistema Único de Saúde, atendendo aproximadamente dois milhões de habitantes em 45 municípios. Conta com 380 leitos ativos, mais de 40 especialidades médicas e serviços de alta complexidade. Como hospital de ensino, integra assistência, ensino, pesquisa e extensão. Destaca-se no que tange à realização de procedimentos cirúrgicos, tendo realizado, no ano de 2019, 7.881 cirurgias, sendo, aproximadamente, 800 do trato digestório^(11)^.

### População e Critérios de Seleção

Participaram pacientes no período pré- ou pós-operatório de cirurgia digestiva, eletiva ou de urgência, internados nas unidades de Clínica Cirúrgica, Clínica Médica e Pronto-Socorro Adulto. O tamanho da amostra foi estimado pelo método de Cochran com correção para população finita, considerando a população de 1.615 (pacientes submetidos a cirurgias digestivas nos dois anos anteriores a outubro de 2023), nível de confiança de 95% (Z = 1,96), margem de erro de 5% e proporção esperada de 50%, obtendo como amostragem mínima o total de 311 participantes. A amostra selecionada foi intencional e de conveniência. Diariamente, membros da equipe de pesquisa rastreavam nas unidades locais do estudo, por meio de informações nos prontuários eletrônicos, os pacientes que se adequavam aos critérios de inclusão/exclusão, constituindo uma lista de possíveis participantes, os quais eram abordados sequencialmente.

Foram incluídos pacientes com idade igual ou superior a 18 anos, hospitalizados nas unidades mencionadas, em período pré- ou pós-operatório de cirurgias digestivas. Foram excluídos aqueles que apresentavam limitações de comunicação e interação, como dificuldade na fala, sonolência excessiva ou instabilidade emocional significativa. A amostra final foi constituída de 319 indivíduos.

### Coleta de Dados

A coleta de dados foi realizada diariamente, de outubro de 2023 a julho de 2024, por uma pesquisadora principal e auxiliares de pesquisa (alunas de pós-graduação), que receberam treinamento prévio teórico-prático. Antes de iniciar a coleta de dados, de forma individual, foi apresentado o estudo, e foram esclarecidas as dúvidas aos possíveis participantes. Foram coletadas assinaturas do Termo de Consentimento Livre e Esclarecido dos que concordaram em participar da pesquisa. Em ambiente reservado, garantindo confidencialidade, o entrevistador aplicou, de forma oral, o formulário de caracterização sociodemográfica e clínica e o *Health Literacy Questionnaire*-versão brasileira (HLQ-Br). Ressalta-se que, conforme mencionado nos critérios de seleção, os participantes responderam à pesquisa independentemente do período operatório (pré- ou pós-), sendo essa uma variável também analisada.

O HLQ foi elaborado por autores australianos, traduzido e adaptado para o português brasileiro, resultando na versão HLQ-Br^(12)^. O HLQ-Br demonstrou boas propriedades psicométricas em diferentes contextos populacionais. Estudos de validação evidenciaram adequada confiabilidade interna, com coeficientes alfa de Cronbach satisfatórios (superiores a 0,70), além de estrutura fatorial consistente com o modelo original^(12)^. Para sua utilização, neste estudo, foi solicitada autorização formal para uso da versão brasileira do instrumento à universidade australiana (e-mail: hl-info@swin.edu.au).

É um instrumento multidimensional, que contém 44 itens, dispostos em nove escalas, que pode ser autoadministrado ou aplicado por meio de entrevistas. O mesmo é dividido em duas partes, sendo que, na primeira parte (escalas 1 a 5), avalia a percepção de competências e recursos para encontrar, compreender, avaliar e usar informações sobre saúde; e as respostas desta parte variam de “discordo totalmente” (1) a “concordo totalmente” (4). Já a segunda parte (escalas 6 a 9) avalia a facilidade de acesso a serviços e cuidados de saúde, e as respostas variam de “sempre difícil” (1) a “sempre fácil” (5)^(13)^. Para facilitar o entendimento das opções de resposta pelos participantes, utilizou-se, quando necessário, uma régua ilustrativa produzida pela pesquisadora.

As escalas avaliadas são: escala 1 – Compreensão e apoio dos profissionais de saúde (quatro itens); escala 2 – Informações suficientes para cuidar da saúde (quatro itens); escala 3 – Cuidado ativo da saúde (cinco itens); escala 4 – Suporte social para saúde (cinco itens); escala 5 – Avaliação das informações em saúde (cinco itens); escala 6 – Capacidade de interagir ativamente com os profissionais de saúde (cinco itens); escala 7 – Navegar no sistema de saúde (seis itens); escala 8 – Capacidade de encontrar boas informações sobre saúde (cinco itens); escala 9 – Compreender as informações sobre saúde e saber o que fazer (cinco itens)^(12)^.

### Variáveis do Estudo

As variáveis dependentes corresponderam às nove escalas do HLQ-Br, tratadas como escores médios contínuos (parte 1: 1–4; parte 2: 1–5). As variáveis independentes incluíram aspectos sociodemográficos (idade, sexo, raça/cor, situação conjugal, escolaridade, renda familiar, possuir filhos, número de residentes no domicílio) e clínicos/assistenciais (presença de acompanhante na internação, comorbidades, uso de dispositivo assistivo, período perioperatório: pré- ou pós-operatório, tempo de internação e tipo de cirurgia).

### Análise dos Dados

Os dados foram analisados por meio do programa *Statistical Package for the Social Sciences*, versão 22. Para caracterização sociodemográfica e clínica, foi utilizada a estatística descritiva. O HLQ-Br não fornece avaliação global para o questionário. Cada escala foi pontuada pela média dos seus itens, sendo que a maior média indicou potencialidade, e a menor, fragilidade do LS^(12)^. A consistência interna foi medida pelo alfa de Cronbach, mostrando-se adequada (α = 0,82 na parte 1 e 0,85 na parte 2).

Para análise bivariada, foram aplicados o teste de Mann-Whitney e o teste de Kruskal-Wallis, seguidos pelas comparações múltiplas de Dunn, uma vez que o teste de normalidade de Shapiro-Wilk apontou dados com distribuição anormal (p < 0,05). Após, as variáveis independentes que apresentaram associação estatisticamente significativa com as escalas do HLQ (p < 0,05) foram selecionadas para a modelagem por regressão linear múltipla. Assim, foram realizadas nove regressões lineares múltiplas, seguindo os pressupostos desse tipo de análise (independência dos resíduos, ausência de multicolinearidade, ausência de outliers, resíduos normalmente distribuídos e homocedasticidade).

### Aspectos Éticos

O estudo foi aprovado pelo Comitê de Ética em Pesquisa da Universidade Federal de Santa Maria, sob Parecer n° 6.423.890, e conduzido conforme as diretrizes éticas estabelecidas pelas Resoluções n° 466/2012 e nº 510/2016 do Conselho Nacional de Saúde. Todos os participantes assinaram o Termo de Consentimento Livre e Esclarecido.

## RESULTADOS

Participaram da pesquisa 319 pacientes, cuja média de idade foi de 53,25 (DP = 17,43) anos. A maioria era do sexo masculino (53,9%), autodeclarada de raça branca (73,7%), possuía companheiro (68,7%) e tinha filhos (68,7%). No que se refere aos aspectos socioeconômicos, 51,4% apresentavam renda familiar de até três salários mínimos, e 59,6% tinham escolaridade até o ensino fundamental. A maioria estava acompanhada durante a hospitalização (90,3%).

Em relação às condições clínicas, 65,5% referiram possuir alguma comorbidade, e 78,4% estavam no período pós-operatório no momento da coleta de dados. Quanto aos procedimentos cirúrgicos realizados pelos participantes, prevaleceram laparotomia (n = 74; 23,2%), apendicectomia (n = 64; 20,1%), colecistectomia (n = 56; 17,6%) e colectomia (n = 15; 4,7%).

Por meio da análise descritiva do HQL-Br, foram identificadas como potencialidades do LS o “Suporte social para saúde”, com média 3,25 ± 0,44, na parte 1, e a “Capacidade de interagir ativamente com os profissionais de saúde”, com média 3,70 ± 0,69, na parte 2. Enquanto isso, as limitações encontradas foram “Informações suficientes para cuidar da saúde”, com média 2,73 ± 0,29, na parte 1, e “Capacidade de encontrar boas informações sobre saúde”, com média 3,39 ± 0,71, na parte 2, conforme descrito na Tabela 1.

**Tabela 1:** Medidas descritivas do *Health Literacy Questionnaire*-versão brasileira de 319 pacientes hospitalizados por cirurgia digestive – Santa Maria, RS, Brasil, 2024.

Escalas do HLQ-Br	Número de itens	Média (DP)^1^	IC95%
**Parte 1**			
1 – Compreensão e apoio dos profissionais de saúde	4	2,94 (0,53)	(2,88–3,00)
2 – Informações suficientes para cuidar da saúde	5	2,73 (0,29)	(2,67–2,79)
3 – Cuidado ativo da saúde	5	2,76 (0,44)	(2,71–2,81)
4 – Suporte social para saúde	5	3,25 (0,44)	(3,20–3,30)
5 – Avaliação das informações em saúde	5	2,82 (0,46)	(2,77–2,87)
**Parte 2**			
6 – Capacidade de interagir ativamente com os profissionais de saúde	5	3,70 (0,69)	(3,63–3,78)
7 – Navegar no sistema de saúde	6	3,42 (0,65)	(3,35–3,49)
8 – Capacidade de encontrar boas informações sobre saúde	5	3,39 (0,71)	(3,31–3,47)
9 – Compreender as informações sobre saúde e saber o que fazer	5	3,58 (0,69)	(3,50–3,66)

Nota: dados apresentados como média (DP – desvio padrão). Valores adicionais entre parênteses na coluna correspondente indicam o Intervalo de Confiança de 95% (IC95%).

Fonte: dados da pesquisa (2024).

As Tabelas 2 e 3 apresentam os resultados da análise bivariada entre as escalas do HLQ-Br e as variáveis sociodemográficas e clínicas dos participantes, correspondendo à parte 1 e à parte 2 do instrumento, respectivamente. Na parte 1, foi identificado que pessoas não brancas, com baixa escolaridade e sem comorbidades relataram menos compreensão e apoio por parte dos profissionais de saúde. Mulheres, com apenas ensino fundamental e aqueles sem doenças pré-existentes também se sentiram menos informados para cuidar da própria saúde.

**Tabela 2:** Análise bivariada da parte 1 do *Health Literacy Questionnaire*-versão brasileira com as variáveis sociodemográficas e clínicas – Santa Maria, RS, Brasil, 2024.

Variáveis	Escalas do HLQ-Br Parte 1 (escores 1–4)				
	Escala 1	Escala 2	Escala 3	Escala 4	Escala 5
**Sexo^+^**					
Feminino	2,91 (0,54)	2,67 (0,56)	2,74 (0,46)	3,20 (0,45)	2,80 (0,43)
Masculino	2,97 (0,54)	2,78 (0,52)	2,78 (0,43)	3,30 (0,44)	2,84 (0,48)
Valor de p	0,248	**0,029**	0,442	**0,042**	0,462
**Raça**					
Branca	2,98 (0,56)	2,73 (0,55)	2,79 (0,44)	3,28 (0,44)	2,84 (0,45)
Não branca	2,83 (0,45)	2,74 (0,52)	2,66 (0,45)	3,16 (0,45)	2,76 (0,49)
Valor de p	**0,003**	0,671	**0,031**	**0,020**	0,191
**Situação conjugal**					
Com companheiro	2,95 (0,49)	2,71 (0,52)	2,77 (0,43)	3,28 (0,41)	2,80 (0,44)
Sem companheiro	2,90 (0,61)	2,76 (0,58)	2,71 (0,45)	3,18 (0,50)	2,84 (0,49)
Valor de p	0,799	0,398	0,163	0,088	0,601
**Escolaridade^#^**					
Analfabeto ou ensino fundamental	2,87 (0,51)^b^	2,64 (0,52)^b^	2,78 (0,42)	3,24 (0,41)^b^	2,70 (0,44)^c^
Ensino médio	2,97 (0,56)^b^	2,83 (0,52)^a^	2,68 (0,47)	3,19 (0,51)^b^	2,93 (0,40)^b^
Ensino superior ou pós-graduação	3,24 (0,50)^a^	2,95 (0,57)^a^	2,83 (0,44)	3,48 (0,34)^a^	3,19 (0,41)^a^
Valor de p	**0,002**	**0,004**	0,066	**0,004**	**< 0,001**
**Possui filhos**					
Sim	2,94 (0,53)	2,71 (0,55)	2,75 (0,46)	3,26 (0,44)	2,80 (0,46)
Não	2,95 (0,58)	2,83 (0,48)	2,79 (0,38)	3,23 (0,49)	2,91 (0,44)
Valor de p	0,519	0,160	0,708	0,894	0,143
**Renda^#^**					
Até um SM	2,84 (0,54)	2,67 (0,56)	2,76 (0,45)	3,14 (0,49)^b^	2,78 (0,47)
Dois ou três SMs	2,95 (0,55)	2,74 (0,54)	2,78 (0,44)	3,27 (0,43)^a^	2,80 (0,45)
> três SMs	3,03 (0,48)	2,78 (0,52)	2,73 (0,44)	3,33 (0,42)^a^	2,92 (0,47)
Valor de p	0,333	0,595	0,577	**0,034**	0,079
**Possui acompanhante no hospital**					
Sim	2,96 (0,51)	2,73 (0,53)	2,74 (0,45)	3,27 (0,42)	2,81 (0,46)
Não	2,77 (0,68)	2,68 (0,58)	2,90 (0,39)	3,00 (0,58)	2,87 (0,47)
Valor de p	0,329	0,997	0,094	**0,010**	0,683
**Possui comorbidades**					
Sim	3,02 (0,48)	2,77 (0,52)	2,80 (0,44)	3,27 (0,44)	2,82 (0,46)
Não	2,79 (0,60)	2,64 (0,56)	2,68 (0,44)	3,22 (0,45)	2,81 (0,47)
Valor de p	**< 0,001**	**0,017**	**0,011**	0,213	0,661
**Período atual**					
Pré-operatório	2,97 (0,58)	2,72 (0,53)	2,86 (0,36)	3,21 (0,44)	2,83 (0,48)
Pós-operatório	2,93 (0,52)	2,73 (0,54)	2,73 (0,64)	3,26 (0,45)	2,82 (0,45)
Valor de p	0,323	0,822	**0,028**	0,273	0,904

Nota: escala 1 – Compreensão e apoio dos profissionais de saúde; escala 2 – Informações suficientes para cuidar da saúde; escala 3 – Cuidado ativo da saúde; escala 4 – Suporte social para saúde; escala 5 – Avaliação das informações em saúde; SM – salário mínimo; +Teste U de Mann-Whitney; #Teste de Kruskal-Wallis; dados apresentados como média (desvio padrão – DP). Valores adicionais entre parênteses na coluna correspondente indicam o Intervalo de Confiança de 95% (IC95%). As letras sobrescritas (a, b, c) indicam diferenças significativas entre os grupos de acordo com comparações múltiplas de Dunn (*p* < 0,05); grupos que não compartilham a mesma letra diferem significativamente.

Fonte: dados da pesquisa (2024).

**Tabela 3:** Análise bivariada da parte 2 do *Health Literacy Questionnaire*-versão brasileira com as variáveis sociodemográficas e clínicas – Santa Maria, RS, Brasil, 2024.

Variáveis	Escalas do HLQ-Br, parte 2 (escores 1–4)			
	Escala 6	Escala 7	Escala 8	Escala 9
**Sexo^+^**				
Feminino	3,68 (0,71)	3,37 (0,73)	3,38 (0,78)	3,63 (0,69)
Masculino	3,73 (0,68)	3,46 (0,57)	3,40 (0,65)	3,54 (0,70)
Valor de p	0,484	0,209	0,900	0,132
**Raça**				
Branca	3,72 (0,67)	3,40 (0,67)	3,41 (0,68)	3,58 (0,63)
Não branca	3,66 (0,76)	3,49 (0,59)	3,33 (0,79)	3,59 (0,85)
Valor de p	0,739	0,254	0,444	0,825
**Situação conjugal**				
Com companheiro	3,66 (0,68)	3,40 (0,61)	3,37 (0,67)	3,56 (0,70)
Sem companheiro	3,78 (0,70)	3,45 (0,72)	3,42 (0,78)	3,62 (0,67)
Valor de p	0,085	0,397	0,354	0,495
**Escolaridade^#^**				
Analfabeto ou ensino fundamental	3,65 (0,67)	3,34 (0,61)^b^	3,17 (0,68)^c^	3,41 (0,72)^b^
Ensino médio	3,72 (0,74)	3,49 (0,69)^a b^	3,64 (0,63)^b^	3,78 (0,58)^a^
Ensino superior ou pós-	3,93 (0,67)	3,66 (0,67)^a^	3,94 (0,48)^a^	3,96 (0,51)^a^
Valor de p	0,078	**0,011**	**< 0,001**	< 0,001
**Possui filhos**				
Sim	3,67 (0,71)	3,38 (0,66)	3,33 (0,70)	3,51 (0,69)
Não	3,88 (0,62)	3,59 (0,58)	3,67 (0,71)	3,93 (0,61)
Valor de p	**0,041**	**0,032**	**0,001**	**< 0,001**
**Renda^#^**				
Até um SM	3,67 (0,68)	3,42 (0,72)	3,26 (0,75)^b^	3,41 (0,78)
Dois ou três SMs	3,69 (0,72)	3,40 (0,64)	3,36 (0,72)^b^	3,62 (0,70)
> três SMs	3,77 (0,65)	3,46 (0,61)	3,61 (0,57)^a^	3,69 (0,54)
Valor de p	0,730	0,793	**0,005**	0,061
**Possui acompanhante no hospital**				
Sim	3,69 (0,70)	3,42 (0,66)	3,37 (0,70)	3,56 (0,70)
Não	3,83 (0,60)	3,43 (0,60)	3,57 (0,78)	3,80 (0,62)
Valor de p	0,337	0,905	0,189	0,109
**Possui comorbidades**				
Sim	3,67 (0,68)	3,44 (0,66)	3,31 (0,70)	3,48 (0,67)
Nenhuma patologia	3,76 (0,73)	3,39 (0,63)	3,55 (0,69)	3,77 (0,70)
Valor de p	0,178	0,276	**0,004**	**0,001**
**Período atual**				
Pré-operatório	3,86 (0,64)	3,47 (0,66)	3,44 (0,77)	3,55 (0,64)
Pós-operatório	3,66 (0,70)	3,40 (0,65)	3,38 (0,69)	3,59 (0,71)
Valor de p	**0,044**	0,345	0,521	0,441

Nota: escala 6 – Capacidade de interagir ativamente com os profissionais de saúde; escala 7 – Navegar no sistema de saúde; escala 8 – Capacidade de encontrar boas informações sobre saúde; escala 9 – Compreender as informações sobre saúde e saber o que fazer; SM – salário mínimo; +Teste U de Mann-Whitney; #Teste de Kruskal-Wallis; dados apresentados como média (desvio padrão – DP). Valores adicionais entre parênteses na coluna correspondente indicam o Intervalo de Confiança de 95% (IC95%). As letras sobrescritas (a, b, c) indicam diferenças significativas entre os grupos de acordo com comparações múltiplas de Dunn (*p* < 0,05); grupos que não compartilham a mesma letra diferem significativamente.

Fonte: dados da pesquisa (2024).

Em relação ao “Cuidado ativo da saúde”, a fragilidade foi mais evidente em pacientes não brancos, sem comorbidades e que estavam no pós-operatório no momento da coleta. Já o “suporte social para saúde” foi menor entre mulheres, indivíduos não brancos, com renda de até um salário mínimo e pacientes internados sem acompanhante. A “Capacidade de encontrar boas informações sobre saúde” foi mais baixa nos participantes com menor nível educacional e nos que utilizavam dispositivos assistivos.

Na parte 2 do HLQ-Br, evidenciou-se que quem possuía filhos exibiu piores escores em todas as escalas. Os dados sobre o nível educacional mostraram que participantes com ensino fundamental foram os mais vulneráveis para “Navegar no sistema de saúde”, “Capacidade de encontrar boas informações sobre saúde” e “Compreender as informações sobre saúde e saber o que fazer”. A renda familiar de até três salários mínimos se associou à maior dificuldade em localizar boas informações, e participantes com comorbidades apresentaram menor aptidão para encontrar e entender informações de saúde. Além disso, quem estava no pós-operatório relatou menor capacidade de interação ativa com profissionais.

Na Tabela 4, estão as correlações entre o HLQ e as variáveis quantitativas investigadas, como idade, tempo de internação e número de pessoas vivendo na residência. Observam-se uma correlação positiva fraca, entre a idade e as escalas 1, 2, 3 e 4, uma correlação negativa fraca, entre as escalas 5 e 8, e uma correlação moderada, com a escala 9. Ainda, observa-se uma correlação negativa fraca entre o tempo de internação e as escalas 3, 6, 8 e 9, e também uma correlação negativa fraca entre o número de residentes no domicílio e as escalas 2, 3 e 6.

**Tabela 4:** Correlação entre as escalas do *Health Literacy Questionnaire*-versão brasileira com as variáveis quantitativas – Santa Maria, RS, Brasil, 2024.

Dimensões do HLQ-Br	Idade (anos)		Tempo de internação (dias)		Número de pessoas vivendo na residência	
	r	Valor de p	r	Valor de p	r	Valor de p
**Parte 1 (escores 1–4)**						
1 – Compreensão e apoio dos profissionais de saúde	0,207**	**< 0,001**	-0,067	0,234	-0,091	0,104
2 – Informações suficientes para cuidar da saúde	0,117*	**0,037**	-0,082	0,147	-0,153*	**0,006**
3 – Cuidado ativo da saúde	0,167**	**0,003**	-0,119*	**0,035**	-0,200*	**< 0,001**
4 – Suporte social para saúde	0,144*	**0,01**	0,031	0,577	-0,005	0,927
5 – Avaliação das informações em saúde	-0,124*	**0,027**	-0,1	0,076	-0,039	0,489
**Parte 2 (escores 1–5)**						
6 – Capacidade de interagir ativamente com os profissionais de saúde	0,022	0,696	-0,130*	**0,021**	-0,116*	**0,038**
7 – Navegar no sistema de saúde	0,099	0,077	-0,062	0,27	-0,065	0,244
8 – Capacidade de encontrar boas informações sobre saúde	-0,238**	**< 0,001**	-0,152*	**0,007**	0,033	0,562
9 – Compreender as informações sobre saúde e saber o que fazer	-0,332**	**< 0,001**	-0,153*	**0,007**	0,066	0,239

r: coeficiente de correlação de Spearman *p < 0,05 e **p < 0,001.

Fonte: dados da pesquisa (2024).

Após ajuste em regressão linear múltipla, a escolaridade e a idade foram fatores significativamente associados à escala “Compreensão e apoio dos profissionais de saúde”. Os participantes com níveis educacionais inferiores ao ensino superior apresentaram escores mais baixos nessa dimensão, incluindo os analfabetos (coef b = –0,87; IC95%: –1,41 a –0,33), os com ensino fundamental (coef b = –0,49; IC95%: –0,69 a –0,30) e os com ensino médio (coef b = –0,28; IC95%: –0,48 a –0,08). Participantes com maior idade tendem a perceber a melhor compreensão e maior apoio profissional (coef b = 0,008; IC95%: –0,004 a 0,012).

Na escala “Informações suficientes para cuidar da saúde”, escolaridade e número de pessoas no domicílio se mostraram associados. Participantes analfabetos (coef b = –0,56; IC95%: –1,11 a –0,01) e com ensino fundamental (coef b = –0,40; IC95%: –0,60 a –0,20) apresentaram menor percepção de acesso à informação. Além disso, o número de moradores no domicílio se associou inversamente a essa percepção (coef b = –0,051; IC95%: –0,100 a –0,001). No que se refere à escala “Cuidado ativo da saúde”, o número de pessoas que residem no domicílio foi o único fator associado (coef b = –0,060; IC95%: –0,101 a –0,018), indicando que residir com mais pessoas esteve relacionado a um menor engajamento ativo no próprio cuidado.

Para a escala “Suporte social para saúde”, sexo, escolaridade, presença de acompanhante e idade foram variáveis associadas. Mulheres apresentaram menor suporte social (coef b = –0,10; IC95%: –0,20 a –0,01), assim como os participantes com ensino fundamental (coef b = –0,29; IC95%: –0,47 a –0,11) e médio (coef b = –0,31; IC95%: –0,49 a –0,13), em comparação aos com ensino superior. Ter um acompanhante esteve associado a um maior suporte (coef b = 0,22; IC95%: 0,06 a 0,38), e a idade mostrou relação positiva com essa dimensão (coef b = 0,004; IC95%: 0,001 a 0,007). A escolaridade foi o único fator associado à escala “Avaliação das informações em saúde”, com menor pontuação entre analfabetos (coef b = –1,00; IC95%: –1,45 a –0,54), participantes com ensino fundamental (coef b = –0,49; IC95%: –0,65 a –0,32) e ensino médio (coef b = –0,26; IC95%: –0,43 a –0,09).

Sobre a parte 2 do instrumento, a escala “Capacidade de interagir ativamente com os profissionais de saúde” se mostrou associada apenas à variável “ter filhos” (coef b = –0,21; IC95%: –0,42 a –0,01). A escolaridade também influenciou a escala “Navegar no sistema de saúde”, com escores mais baixos entre aqueles com ensino fundamental (coef b = –0,29; IC95%: –0,53 a –0,04). Por fim, na escala “Capacidade de encontrar boas informações sobre saúde”, os participantes analfabetos (coef b = –0,96; IC95%: –1,68 a –0,24) e com ensino fundamental (coef b = –0,64; IC95%: –0,91 a –0,37) também apresentaram menor pontuação. Na escala “Compreender as informações sobre saúde e saber o que fazer”, foram observadas menores pontuações entre analfabetos (coef b = –1,29; IC95%: –1,97 a –0,61) e participantes com ensino fundamental (coef b = –0,38; IC95%: –0,63 a –0,14), além de uma associação inversa com a idade (coef b = –0,004; IC95%: –0,011 a –0,001).

## DISCUSSÃO

Este estudo identificou, como potencialidades do LS, a interação com profissionais e suporte social, e como fragilidades, a insuficiência de informações para o autocuidado e dificuldade em encontrar informações de qualidade. A análise multivariada evidenciou que a escolaridade foi o principal fator associado aos diferentes domínios do HLQ-Br, reforçando a influência do gradiente educacional sobre o LS. Variáveis como idade, presença de filhos, número de moradores no domicílio e comorbidades também apresentaram associações relevantes, revelando nuances importantes para a compreensão da vulnerabilidade dos pacientes cirúrgicos.

A estrutura multidimensional do HLQ-Br permite uma avaliação abrangente, que transcende a análise da mera capacidade de leitura e interpretação textual, incorporando aspectos essenciais como navegação no sistema de saúde, interação com profissionais e percepção do suporte social. Tal característica possibilita identificar, com precisão, áreas de competência já consolidadas, como a compreensão adequada de informações de saúde, a capacidade de localizar fontes confiáveis, a interação efetiva com profissionais, a navegação organizada pelos serviços e o uso de redes de apoio, e, simultaneamente, fragilidades que demandam o direcionamento de intervenções sensíveis às particularidades socioeconômicas e culturais dos pacientes^(14)^.

A análise das escalas apontou como potencialidades do LS, ou seja, aspectos que estão fortalecidos, o “Suporte social para saúde” e a “Capacidade de interagir ativamente com os profissionais de saúde”, sinalizando que as redes de apoio, tanto familiares quanto institucionais, podem exercer um papel protetivo diante dos desafios impostos pela hospitalização. Evidências robustas indicam que indivíduos que se percebem apoiados tendem a apresentar melhor adesão terapêutica, maior engajamento no autocuidado e redução no risco de complicações^(15,16)^, reafirmando o impacto positivo do suporte social sobre os desfechos em saúde.

As maiores limitações do LS identificadas nos pacientes deste estudo foram “Informações suficientes para cuidar da saúde” e “Capacidade de encontrar boas informações sobre saúde”, indicando dificuldades relevantes no acesso e uso de informações para o autocuidado. Esse cenário é especialmente crítico no contexto das cirurgias digestivas, que exigem mudanças complexas no estilo de vida e vigilância rigorosa de complicações. Dessa forma, isso pode ser mais um fator contribuinte para aumentar a escassez de informações no período pós-operatório, dificultando a identificação de falhas no cuidado. Medidas clínicas tradicionais não capturam plenamente a experiência do paciente, limitando a avaliação da qualidade centrada na pessoa^(17)^.

A análise multivariada confirmou que a escolaridade foi a variável mais fortemente associada às dimensões do HLQ-Br, mesmo após ajustes para outros fatores, corroborando evidências de que a baixa escolaridade constitui um importante preditor de menores níveis de LS^(18,19)^. Pacientes com ensino fundamental apresentaram, de forma consistente, os piores escores em diversas dimensões, seguidos por aqueles com ensino médio, enquanto os com ensino superior ou pós-graduação demonstraram melhores desempenhos. Esse gradiente educacional evidencia a relação entre LS e iniquidades sociais, na medida em que limita o acesso, a compreensão e o uso de informações em saúde^(20)^. Tais achados estão alinhados a estudos prévios, os quais descrevem que déficits educacionais podem repercutir negativamente em aspectos como autonomia e tomada de decisão informada, embora esses desfechos não tenham sido avaliados diretamente neste estudo^(18,19)^.

A análise sociodemográfica acrescentou elementos importantes, ainda que não conclusivos. Embora a literatura frequentemente relacione idade avançada a menores níveis de LS, neste estudo, observou-se que pacientes mais velhos tenderam a apresentar escores mais elevados em dimensões vinculadas ao suporte social e ao apoio dos profissionais de saúde, sugerindo que fatores contextuais podem atenuar ou modificar esse padrão. Este achado é corroborado por estudo^(21)^ que destaca o papel do suporte social como mediador positivo da relação entre envelhecimento e engajamento no autocuidado, mitigando, em parte, as limitações associadas à idade.

Outro aspecto que merece atenção refere-se aos menores escores médios identificados entre pacientes que possuíam filhos, sobretudo nas escalas de interação com profissionais, navegação no sistema de saúde e compreensão das informações, sugerindo maior vulnerabilidade nessas dimensões do LS. Esse resultado pode estar relacionado à sobrecarga de responsabilidades familiares, que frequentemente recai sobre esses indivíduos e pode reduzir o tempo, a atenção e os recursos cognitivos dedicados às próprias demandas de saúde. Tal interpretação encontra respaldo na literatura, que associa o acúmulo de papéis familiares e, em especial, as desigualdades de gênero a uma menor capacidade de engajamento em práticas de autocuidado e de busca por informações em saúde^(22,23)^.

Particularmente relevante foi o achado de que pacientes no pós-operatório apresentaram fragilidade acentuada no LS no que diz respeito ao “Cuidado ativo da saúde” e “Capacidade de interagir ativamente com os profissionais de saúde”, achado consistente com a literatura internacional^(15,16)^. Esse achado reforça a necessidade de que a equipe de enfermagem perioperatória incorpore, de forma sistemática, a avaliação do LS ao planejamento do cuidado, a fim de prevenir falhas de comunicação e apoiar a adesão às recomendações clínicas.

A dimensão “Capacidade de encontrar boas informações sobre saúde”, que apresentou uma das médias mais baixas no presente estudo, merece especial atenção, sobretudo diante do contexto contemporâneo de ampla circulação de informações muitas vezes imprecisas, incompletas ou incorretas. Esse cenário expõe os pacientes com baixo LS ao risco de desinformação, adoção de condutas inseguras, uso inadequado de terapias e maior probabilidade de desfechos adversos^(24,25)^.

Diante desse panorama, torna-se evidente a necessidade de que os serviços de saúde adotem estratégias sistemáticas, contínuas e estruturadas para avaliar e fortalecer o LS dos pacientes cirúrgicos. Para garantir segurança, autonomia e adesão ao cuidado, são fundamentais a adoção de práticas como comunicação clara, uso de materiais educativos visuais e validação da compreensão por meio de metodologias como o *teach-back*, além de apoio educacional longitudinal^(26,27)^.

Nesta lógica, a fim de letrar os pacientes, a atuação do enfermeiro no contexto do paciente cirúrgico pode se beneficiar, sobretudo, do método *teach-back*, estratégia comunicacional que favorece a compreensão, a adesão e a segurança do cuidado^(28)^. No pré-operatório, o *teach-back* permite verificar se o paciente entendeu corretamente as instruções sobre jejum, higiene, uso de medicamentos e preparo emocional para a cirurgia. Já no pós-operatório, essa técnica auxilia na explicação dos cuidados com feridas, administração de medicamentos, reconhecimento de sinais de complicações e orientações para o retorno às atividades diárias. Ao pedir que o paciente repita as informações com suas próprias palavras, o enfermeiro confirma o entendimento e identifica possíveis dúvidas, promovendo segurança, autonomia e adesão ao tratamento, além de contribuir para uma recuperação mais rápida e eficaz.

Adicionalmente, os achados reforçam o papel estratégico da equipe de enfermagem, que, pela proximidade com os pacientes, ocupa posição privilegiada na identificação de barreiras ao LS e no desenvolvimento de intervenções educativas, colaborando diretamente para a qualificação do cuidado e a redução de riscos no pós-operatório^(29)^.

Por fim, destaca-se que, apesar da crescente valorização internacional do LS como componente central das práticas de cuidado, ainda há lacunas na produção de conhecimento sobre o tema no contexto brasileiro, especialmente em populações cirúrgicas, o que limita o desenvolvimento de políticas e práticas baseadas em evidências.

Este estudo apresenta algumas limitações, sendo uma delas relacionada ao delineamento transversal, que não permite estabelecer relações de causalidade, restringindo-se à identificação de associações. Menciona-se, ainda, a amostra por conveniência de um único hospital de ensino, limitando a representatividade e a generalização dos achados. Embora o HLQ-Br seja um instrumento validado no Brasil, não dispõe de pontos de corte estabelecidos que permitam a categorização do nível de LS, restringindo a interpretação dos resultados a escores médios. Ademais, a utilização de recursos visuais como apoio à compreensão dos itens representou uma adaptação metodológica que pode ter influenciado as respostas. Destaca-se ainda a possibilidade de viés de seleção, pelo não recrutamento de pacientes com limitações de comunicação, e de viés de informação, uma vez que parte dos dados foi autorreferida. Apesar do controle de confundimento por regressão múltipla, não se pode descartar a influência de variáveis não mensuradas.

Apesar das limitações, este estudo traz contribuições relevantes. Na assistência, subsidia a equipe de enfermagem perioperatória na identificação de pacientes mais vulneráveis em relação ao LS e no planejamento de intervenções educativas individualizadas, com linguagem clara, *teach-back* e materiais adaptados. Na gestão, apoia a incorporação da avaliação do LS como indicador de qualidade e segurança hospitalar, orientando políticas institucionais e programas de capacitação. Na pesquisa, amplia as evidências nacionais sobre LS em pacientes cirúrgicos, estimulando estudos multicêntricos e longitudinais. Por fim, favorece o desenvolvimento de práticas personalizadas, direcionadas conforme o perfil sociodemográfico e clínico, como estratégias diferenciadas para indivíduos com baixa escolaridade, reforço do suporte social em pacientes sem acompanhante e maior acompanhamento no pós-operatório para grupos vulneráveis.

## CONCLUSÃO

O estudo identificou como potencialidades do LS a interação com profissionais e o suporte social. Como fragilidades, foram identificadas a insuficiência de informações para o autocuidado e a dificuldade em encontrar informações de qualidade no contexto de pacientes cirúrgicos em período pré- ou pós-operatório.

A análise multivariada evidenciou que a menor escolaridade foi o principal fator associado a piores escores em diversas dimensões, enquanto variáveis como idade, presença de filhos, número de moradores no domicílio, comorbidades e período perioperatório também apresentaram associações específicas.

Esses achados demonstram a influência do gradiente educacional e de condições sociais sobre o LS, reforçando a importância de incorporar sua avaliação sistemática à prática da enfermagem perioperatória e de desenvolver intervenções educativas acessíveis e centradas no paciente.

## DISPONIBILIDADE DE DADOS

Todo o conjunto de dados que dá suporte aos resultados deste estudo está disponível mediante solicitação ao autor correspondente.

# Translation: Alfabetización en salud de pacientes hospitalizados por cirugías digestivas*

**DOI:** 10.1590/1980-220X-REEUSP-2025-0267es

**Authors:** Stéfani Rodrigues Venturini, Etiane de Oliveira Freitas, Silviamar Camponogara, Nara Marilene Oliveira Girardon-Perlini, Rosângela Marion da Silva, Liana Amorim Côrrea Trotte, Larissa Venturini

**Affiliations:** 1 Universidade Federal de Santa Maria, Departamento de Enfermagem, Santa Maria, RS, Brasil.; 2 Universidade Federal do Rio de Janeiro, Departamento de Enfermagem, Rio de Janeiro, RJ, Brasil.; 3 Universidade Federal de Santa Maria, Hospital Universitário de Santa Maria, Santa Maria, RS, Brasil.

**Keywords:** Alfabetización en Salud, Pacientes Internos, Procedimientos Quirúrgicos Operativos, Sistema Digestivo, Enfermería Perioperatoria

## Abstract

**Objetivos:**

Evaluar la alfabetización en salud (AS) de pacientes en el período preoperatorio y posoperatorio de cirugías digestivas y verificar su asociación con variables sociodemográficas y clínicas.

**Método:**

Estudio transversal realizado en un hospital público. Los datos se recopilaron entre octubre de 2023 y julio de 2024 mediante un cuestionario con datos sociodemográficos y clínicos, y el Health Literacy Questionnaire (HLQ). Se realizaron análisis descriptivos, pruebas estadísticas inferenciales y regresión lineal múltiple.

**Resultados:**

De los 319 participantes, el 78,4% se encontraba en el período postoperatorio al momento de la recolección de datos. Entre las escalas del HLQ, la “Capacidad para interactuar activamente con profesionales de la salud” obtuvo la mejor calificación (3,70 ± 0,69), mientras que la “Información suficiente para cuidar la propia salud” (2,73 ± 0,29) obtuvo la peor calificación. El nivel educativo, la raza, la edad, tener hijos, vivir con otras personas y contar con un cuidador se asociaron con la AS (p < 0,05). En el análisis ajustado, las variables permanecieron asociadas, excepto la raza, los ingresos y el período operatorio.

**Conclusión:**

La interacción con profesionales contribuye a la AS, mientras que la comprensión y la evaluación crítica de la información sanitaria la debilitan. Un nivel educativo bajo fue el principal factor asociado a una menor AS.

## INTRODUCCIÓN

La alfabetización en salud (AS) surge como un determinante social crítico reconocido por la Organización Mundial de la Salud como un elemento central para la prevención, el control y el manejo de las enfermedades crónicas no transmisibles a lo largo de la vida^(1)^. La definición más reciente del término AS fue presentada por la iniciativa Health People 2030, en Estados Unidos, que lo describe como la capacidad que tienen los individuos de encontrar, comprender y utilizar información y servicios para tomar decisiones y acciones relacionadas con la salud para sí mismos y para los demás^(2)^. Cuando es insuficiente, se asocia con una menor adherencia a las recomendaciones terapéuticas, dificultad para interpretar las recetas, uso inadecuado de medicamentos y barreras en el desarrollo de prácticas de autocuidado, lo que repercute negativamente en los resultados clínicos y la seguridad del paciente^(1,3)^. Estos aspectos cobran especial relevancia en el contexto quirúrgico, en el que el paciente necesita comprender orientaciones complejas relacionadas con la preparación preoperatoria, el autocuidado en el postoperatorio y la prevención de complicaciones.

La evaluación de la AS, por lo tanto, se configura como una estrategia esencial para hacer frente a las desigualdades en salud y en la calificación de los procesos de atención, especialmente en contextos que exigen un alto grado de compromiso por parte del paciente^(2,3)^, como el periodo pre y postoperatorio de las cirugías digestivas. Las cirugías digestivas, frecuentemente indicadas en el manejo de afecciones complejas del tracto gastrointestinal, como neoplasias, enfermedad por reflujo gastroesofágico, colelitiasis, pancreatitis, hernias abdominales y síndromes intestinales, exigen que los pacientes comprendan los aspectos clínicos y las adaptaciones conductuales y psicosociales^(4,5)^. El post operatorio, en particular, impone cambios sustanciales en el estilo de vida, como modificaciones en la dieta, ajustes en la rutina, manejo de dispositivos, como ostomías, sondas y drenajes, además de afrontar los impactos físicos y emocionales derivados de la intervención quirúrgica^(6,7)^.

La literatura internacional señala que la limitación de la AS es un problema frecuente entre los pacientes quirúrgicos. Un metaanálisis de 40 estudios, con un total de 18 895 individuos, estimó que aproximadamente un tercio de esta población presenta un AS inadecuado (31,7%)^(8)^.

Las evidencias convergentes indican que este déficit se asocia a resultados clínicos desfavorables, como una menor adherencia a las orientaciones pre y postoperatorias, un mayor tiempo de hospitalización, un aumento de las tasas de reingreso y un incremento de los costos hospitalarios^(9)^. Corroborando estos hallazgos, un estudio retrospectivo reciente con pacientes sometidos a cirugía colorrectal electiva identificó que la AS limitada duplicaba el riesgo de complicaciones postoperatorias, aumentaba la incidencia de infección del sitio quirúrgico y prolongaba la estancia hospitalaria^(10)^. Aunque la revisión de Roy et al.^(8)^ ha consolidado la evidencia sobre la AS en poblaciones quirúrgicas, los autores destacan la escasez de investigaciones realizadas en países de América Latina, incluido el Brasil, lo que limita la comprensión de la realidad local.

A pesar del reconocimiento mundial de la AS como determinante crítico de la seguridad quirúrgica, en Brasil persisten lagunas sustanciales, ya que no se han identificado investigaciones aplicadas a pacientes quirúrgicos en revisiones internacionales^(8)^. Las investigaciones nacionales en enfermería perioperatoria abordan aspectos como las complicaciones postoperatorias^(4)^, la evolución nutricional^(5)^ y la adaptación a los dispositivos quirúrgicos^(7)^, pero aún no han explorado directamente la dimensión de la AS en este grupo.

Así, este estudio añade evidencia inédita al cuantificar la AS de pacientes hospitalizados en el periodo preoperatorio o postoperatorio de cirugías digestivas en Brasil, contribuyendo a fundamentar estrategias de cuidado orientadas por evidencia científica, además de potenciar la calidad y la equidad de los servicios de salud. Ante este panorama, el objetivo de este estudio es evaluar la AS de pacientes en el periodo preoperatorio o postoperatorio de cirugías digestivas y verificar las asociaciones con variables sociodemográficas y clínicas.

## MÉTODO

### Diseño del Estudio

Se trata de un estudio transversal, diseñado según las directrices de *STrengthening the Reporting of OBservational studies in Epidemiology*.

### Entorno

Se trata de un hospital público y universitario, ubicado en la región central de Rio Grande do Sul, que es una referencia regional en atención de media y alta complejidad por el Sistema Único de Salud, atendiendo a aproximadamente dos millones de habitantes en 45 municipios. Cuenta con 380 camas activas, más de 40 especialidades médicas y servicios de alta complejidad. Como hospital universitario, integra la asistencia, la enseñanza, la investigación y la extensión. Destaca en la realización de procedimientos quirúrgicos, habiendo realizado, en el año 2019, 7881 cirugías, de las cuales aproximadamente 800 fueron del tracto digestivo^(11)^.

### Población y Criterios de Selección

Participaron pacientes en el periodo preoperatorio o postoperatorio de cirugía digestiva, electiva o de urgencia, ingresados en las unidades de Cirugía, Medicina y Urgencias para adultos.

El tamaño de la muestra se estimó mediante el método de Cochran con corrección para población finita, considerando una población de 1615 (pacientes sometidos a cirugías digestivas en los dos años anteriores a octubre de 2023), un nivel de confianza del 95% (Z = 1,96), margen de error del 5% y proporción esperada del 50%, obteniendo como muestra mínima un total de 311 participantes. La muestra seleccionada fue intencional y por conveniencia. Diariamente, los miembros del equipo de investigación rastreaban en las unidades locales del estudio, a través de la información de los expedientes médicos electrónicos, a los pacientes que se ajustaban a los criterios de inclusión/exclusión, constituyendo una lista de posibles participantes, a los que se abordaba secuencialmente.

Se incluyeron pacientes de 18 años o más, hospitalizados en las unidades mencionadas, en el periodo preoperatorio o postoperatorio de cirugías digestivas. Se excluyó a aquellos que presentaban limitaciones de comunicación e interacción, como dificultad para hablar, somnolencia excesiva o inestabilidad emocional significativa. La muestra final estuvo compuesta por 319 individuos.

### Recolección de Datos

La recolección de datos se realizó diariamente, de octubre de 2023 a julio de 2024, por una investigadora principal y auxiliares de investigación (estudiantes de posgrado), que recibieron formación teórica y práctica previa. Antes de iniciar la recolección de datos, de forma individual, se presentó el estudio y se aclararon las dudas a los posibles participantes. Se recabaron las firmas del Término de Consentimiento Libre e Informado de quienes aceptaron participar en la investigación. En un entorno reservado, garantizando la confidencialidad, el entrevistador aplicó, de forma oral, el formulario de caracterización sociodemográfica y clínica y el Health Literacy Questionnaire (Cuestionario de Alfabetización en Salud), versión brasileña (HLQ-Br). Cabe destacar que, tal y como se menciona en los criterios de selección, los participantes respondieron a la encuesta independientemente del periodo operatorio (previo o posterior), siendo esta una variable también analizada.

El HLQ fue elaborado por autores australianos, traducido y adaptado al portugués brasileño, dando como resultado la versión HLQ-Br^(12)^. El HLQ-Br demostró buenas propiedades psicométricas en diferentes contextos poblacionales. Los estudios de validación evidenciaron una fiabilidad interna adecuada, con coeficientes alfa de Cronbach satisfactorios (superiores a 0,70), además de una estructura factorial consistente con el modelo original^(12)^. Para su uso en este estudio, se solicitó autorización formal a la universidad australiana (correo electrónico: hl-info@swin.edu.au) para utilizar la versión brasileña del instrumento.

Es un instrumento multidimensional que contiene 44 ítems, dispuestos en nueve escalas, que puede ser autoadministrado o aplicado mediante entrevistas. Se divide en dos partes: la primera (escalas 1 a 5) evalúa la percepción de las competencias y los recursos para encontrar, comprender, evaluar y utilizar información sobre salud; las respuestas de esta parte varían de “totalmente en desacuerdo” (1) a “totalmente de acuerdo” (4). La segunda parte (escalas 6 a 9) evalúa la facilidad de acceso a los servicios y la atención sanitaria, y las respuestas varían de “siempre difícil” (1) a “siempre fácil” (5)^(13)^. Para facilitar la comprensión de las opciones de respuesta por parte de los participantes, se utilizó, cuando fue necesario, una regla ilustrativa elaborada por la investigadora.

Las escalas evaluadas son: escala 1 – Comprensión y apoyo de los profesionales de la salud (cuatro ítems); escala 2 – Información suficiente para cuidar la salud (cuatro ítems); escala 3 – Cuidado activo de la salud (cinco ítems); escala 4 – Apoyo social para la salud (cinco ítems); escala 5 – Evaluación de la información en salud (cinco ítems); escala 6 – Capacidad para interactuar activamente con los profesionales de la salud (cinco ítems); escala 7 – Navegar por el sistema de salud (seis ítems); escala 8 – Capacidad para encontrar buena información en salud (cinco ítems); escala 9 – Comprensión de la información sobre salud y saber qué hacer (cinco ítems)^(12)^.

### Variables del Estudio

Las variables dependientes correspondieron a las nueve escalas del HLQ-Br, tratadas como puntuaciones medias continuas (parte 1: 1–4; parte 2: 1–5). Las variables independientes incluyeron aspectos sociodemográficos (edad, sexo, raza/color, estado civil, nivel educativo, ingresos familiares, tener hijos, número de residentes en el domicilio) y clínicos/asistenciales (presencia de acompañante durante la hospitalización, comorbilidades, uso de dispositivos de asistencia, periodo perioperatorio: preoperatorio o postoperatorio, duración de la hospitalización y tipo de cirugía).

### Análisis de los Datos

Los datos se analizaron mediante el programa Statistical Package for the Social Sciences, versión 22. Para la caracterización sociodemográfica y clínica, se utilizó la estadística descriptiva. El HLQ-Br no proporciona una evaluación global del cuestionario.

Cada escala se puntuó mediante la media de sus ítems, indicando la media más alta el potencial y la más baja la fragilidad de la AS^(12)^. La consistencia interna se midió mediante el alfa de Cronbach, resultando adecuada (α = 0,82 en la parte 1 y 0,85 en la parte 2).

Para el análisis bivariado, se aplicaron la prueba de Mann-Whitney y la prueba de Kruskal-Wallis, seguidas de las comparaciones múltiples de Dunn, ya que la prueba de normalidad de Shapiro-Wilk indicó datos con distribución anormal (p < 0,05). A continuación, se seleccionaron las variables independientes que presentaban una asociación estadísticamente significativa con las escalas del HLQ (p < 0,05) para el modelado por regresión lineal múltiple. Así, se realizaron nueve regresiones lineales múltiples, siguiendo los supuestos de este tipo de análisis (independencia de los residuos, ausencia de multicolinealidad, ausencia de valores atípicos, residuos normalmente distribuidos y homocedasticidad).

### Aspectos Éticos

El estudio fue aprobado por el Comité de Ética en Investigación de la Universidad Federal de Santa María, bajo el dictamen n.º 6.423.890, y se llevó a cabo de acuerdo con las directrices éticas establecidas por las Resoluciones n.º 466/2012 y n.º 510/2016 del Consejo Nacional de Salud. Todos los participantes firmaron el Término de Consentimiento Libre e Ilustrado.

## RESULTADOS

Participaron en la investigación 319 pacientes, cuya edad media era de 53,25 (DE = 17,43) años. La mayoría eran hombres (53,9%), se autodeclaraban de raza blanca (73,7%), tenían pareja (68,7%) e hijos (68,7%). En cuanto a los aspectos socioeconómicos, el 51,4% tenía ingresos familiares de hasta tres salarios mínimos y el 59,6% tenía estudios de primaria. La mayoría estaba acompañada durante la hospitalización (90,3%).

En cuanto a las condiciones clínicas, el 65,5% refirió tener alguna comorbilidad y el 78,4% se encontraba en el periodo postoperatorio en el momento de la recolección de datos. En cuanto a los procedimientos quirúrgicos realizados a los participantes, predominaron la laparotomía (n = 74; 23,2%), la apendicectomía (n = 64; 20,1%), la colecistectomía (n = 56; 17,6%) y la colectomía (n = 15; 4,7%).

Mediante el análisis descriptivo del HQL-Br, se identificaron como potencialidades de la AS el “Apoyo social para la salud”, con una media de 3,25 ± 0,44, en la parte 1, y la “Capacidad de interactuar activamente con los profesionales de la salud”, con una media de 3,70 ± 0,69, en la parte 2. Por su parte, las limitaciones encontradas fueron “Información suficiente para cuidar la salud”, con una media de 2,73 ± 0,29, en la parte 1, y “Capacidad para encontrar buena información sobre salud”, con una media de 3,39 ± 0,71, en la parte 2, tal y como se describe en la Tabla 1.

**Tabla 1:** Medidas descriptivas del *Health Literacy Questionnaire* (Cuestionario de Alfabetización en Salud), versión brasileña, de 319 pacientes hospitalizados por cirugía digestive – Santa María, RS, Brasil, 2024.

Escalas del HLQ-Br	Número de ítems	Media (DE)^1^	IC95%
**Parte 1**			
1 – Comprensión y apoyo de los profesionales de la salud	4	2,94 (0,53)	(2,88–3,00)
2 – Información suficiente para cuidar de la salud	5	2,73 (0,29)	(2,67–2,79)
3 – Cuidado activo de la salud	5	2,76 (0,44)	(2,71–2,81)
4 – Apoyo social para la salud	5	3,25 (0,44)	(3,20–3,30)
5 – Evaluación de la información en salud	5	2,82 (0,46)	(2,77–2,87)
**Parte 2**			
6 – Capacidad de interactuar activamente con los profesionales de salud	5	3,70 (0,69)	(3,63–3,78)
7 – Navegar en el sistema de salud	6	3,42 (0,65)	(3,35–3,49)
8 – Capacidad de encontrar buenas informaciones sobre salud	5	3,39 (0,71)	(3,31–3,47)
9 – Comprender las informaciones sobre salud y saber que hacer	5	3,58 (0,69)	(3,50–3,66)

Nota: datos presentados como media (DE – desviación estándar). Los valores adicionales entre paréntesis en la columna correspondiente indican el intervalo de confianza del 95% (IC95%).

Fuente: datos de la investigación (2024).

Las Tablas 2 y 3 presentan los resultados del análisis bivariado entre las escalas del HLQ-Br y las variables sociodemográficas y clínicas de los participantes, correspondientes a la parte 1 y la parte 2 del instrumento, respectivamente. En la parte 1, se identificó que las personas no blancas, con bajo nivel educativo y sin comorbilidades informaron menos comprensión y apoyo por parte de los profesionales de la salud. Las mujeres, con solo educación primaria y aquellas sin enfermedades preexistentes también se sintieron menos informadas para cuidar de su propia salud.

**Tabla 2:** Análisis bivariado de la parte 1 del *Health Literacy Questionnaire* (Cuestionario de Alfabetización en Salud), versión brasileña, con las variables sociodemográficas y clínicas – Santa María, RS, Brasil, 2024.

Variables	Escalas del HLQ-Br Parte 1 (escores 1–4)				
	Escala 1	Escala 2	Escala 3	Escala 4	Escala 5
**Sexo^+^**					
Femenino	2,91 (0,54)	2,67 (0,56)	2,74 (0,46)	3,20 (0,45)	2,80 (0,43)
Masculino	2,97 (0,54)	2,78 (0,52)	2,78 (0,43)	3,30 (0,44)	2,84 (0,48)
Valor de p	0,248	**0,029**	0,442	**0,042**	0,462
**Raza**					
Blanca	2,98 (0,56)	2,73 (0,55)	2,79 (0,44)	3,28 (0,44)	2,84 (0,45)
No blanca	2,83 (0,45)	2,74 (0,52)	2,66 (0,45)	3,16 (0,45)	2,76 (0,49)
Valor de p	**0,003**	0,671	**0,031**	**0,020**	0,191
**Estado conyugal**					
Con compañero	2,95 (0,49)	2,71 (0,52)	2,77 (0,43)	3,28 (0,41)	2,80 (0,44)
Sin compañero	2,90 (0,61)	2,76 (0,58)	2,71 (0,45)	3,18 (0,50)	2,84 (0,49)
Valor de p	0,799	0,398	0,163	0,088	0,601
**Escolaridad^#^**					
Analfabeto o primaria	2,87 (0,51)^b^	2,64 (0,52)^b^	2,78 (0,42)	3,24 (0,41)^b^	2,70 (0,44)^c^
Secundaria	2,97 (0,56)^b^	2,83 (0,52)^a^	2,68 (0,47)	3,19 (0,51)^b^	2,93 (0,40)^b^
Superior o postgrado	3,24 (0,50)^a^	2,95 (0,57)^a^	2,83 (0,44)	3,48 (0,34)^a^	3,19 (0,41)^a^
Valor de p	**0,002**	**0,004**	0,066	**0,004**	**< 0,001**
**Hijos**					
Si	2,94 (0,53)	2,71 (0,55)	2,75 (0,46)	3,26 (0,44)	2,80 (0,46)
No	2,95 (0,58)	2,83 (0,48)	2,79 (0,38)	3,23 (0,49)	2,91 (0,44)
Valor de p	0,519	0,160	0,708	0,894	0,143
**Ingreso^#^**					
Hasta 1 SM	2,84 (0,54)	2,67 (0,56)	2,76 (0,45)	3,14 (0,49)^b^	2,78 (0,47)
2 a 3 SMs	2,95 (0,55)	2,74 (0,54)	2,78 (0,44)	3,27 (0,43)^a^	2,80 (0,45)
> tres SMs	3,03 (0,48)	2,78 (0,52)	2,73 (0,44)	3,33 (0,42)^a^	2,92 (0,47)
Valor de p	0,333	0,595	0,577	**0,034**	0,079
**Tiene acompañante en el hospital**					
Si	2,96 (0,51)	2,73 (0,53)	2,74 (0,45)	3,27 (0,42)	2,81 (0,46)
No	2,77 (0,68)	2,68 (0,58)	2,90 (0,39)	3,00 (0,58)	2,87 (0,47)
Valor de p	0,329	0,997	0,094	**0,010**	0,683
**Tiene comorbilidades**					
Si	3,02 (0,48)	2,77 (0,52)	2,80 (0,44)	3,27 (0,44)	2,82 (0,46)
No	2,79 (0,60)	2,64 (0,56)	2,68 (0,44)	3,22 (0,45)	2,81 (0,47)
Valor de p	**< 0,001**	**0,017**	**0,011**	0,213	0,661
**Período actual**					
Pre-operatorio	2,97 (0,58)	2,72 (0,53)	2,86 (0,36)	3,21 (0,44)	2,83 (0,48)
Post-operatorio	2,93 (0,52)	2,73 (0,54)	2,73 (0,64)	3,26 (0,45)	2,82 (0,45)
Valor de p	0,323	0,822	**0,028**	0,273	0,904

Nota: escala 1 – Comprensión y apoyo de los profesionales de la salud; escala 2 – Información suficiente para cuidar la salud; escala 3 – Cuidado activo de la salud; escala 4 – Apoyo social para la salud; escala 5 – Evaluación de la información en salud; SM – salario mínimo; +Prueba U de Mann-Whitney; #Prueba de Kruskal-Wallis; datos presentados como media (desviación estándar – DE). Los valores adicionales entre paréntesis en la columna correspondiente indican el intervalo de confianza del 95% (IC95%). Las letras superpuestas (a, b, c) indican diferencias significativas entre los grupos según las comparaciones múltiples de Dunn (p < 0,05); los grupos que no comparten la misma letra difieren significativamente.

Fuente: datos de la investigación (2024).

**Tabla 3:** Análisis bivariado de la parte 2 del *Health Literacy Questionnaire* (Cuestionario de Alfabetización en Salud), versión brasileña, con las variables sociodemográficas y clínicas – Santa María, RS, Brasil, 2024.

Variables	Escalas del HLQ-Br, parte 2 (escores 1-4)			
	Escala 6	Escala 7	Escala 8	Escala 9
**Sexo^+^**				
Femenino	3,68 (0,71)	3,37 (0,73)	3,38 (0,78)	3,63 (0,69)
Masculino	3,73 (0,68)	3,46 (0,57)	3,40 (0,65)	3,54 (0,70)
Valor de p	0,484	0,209	0,900	0,132
**Raza**				
Blanca	3,72 (0,67)	3,40 (0,67)	3,41 (0,68)	3,58 (0,63)
No blanca	3,66 (0,76)	3,49 (0,59)	3,33 (0,79)	3,59 (0,85)
Valor de p	0,739	0,254	0,444	0,825
**Estado conyugal**				
Con compañero	3,66 (0,68)	3,40 (0,61)	3,37 (0,67)	3,56 (0,70)
Sin compañero	3,78 (0,70)	3,45 (0,72)	3,42 (0,78)	3,62 (0,67)
Valor de p	0,085	0,397	0,354	0,495
**Escolaridad^#^**				
Analfabeto o primaria	3,65 (0,67)	3,34 (0,61)^b^	3,17 (0,68)^c^	3,41 (0,72)^b^
Secundaria	3,72 (0,74)	3,49 (0,69)^a b^	3,64 (0,63)^b^	3,78 (0,58)^a^
Superior o postgrado	3,93 (0,67)	3,66 (0,67)^a^	3,94 (0,48)^a^	3,96 (0,51)^a^
Valor de p	0,078	**0,011**	**< 0,001**	< 0,001
**Hijos**				
Si	3,67 (0,71)	3,38 (0,66)	3,33 (0,70)	3,51 (0,69)
No	3,88 (0,62)	3,59 (0,58)	3,67 (0,71)	3,93 (0,61)
Valor de p	**0,041**	**0,032**	**0,001**	**< 0,001**
**Ingreso^#^**				
Hasta 1 SM	3,67 (0,68)	3,42 (0,72)	3,26 (0,75)^b^	3,41 (0,78)
2 a 3 SMs	3,69 (0,72)	3,40 (0,64)	3,36 (0,72)^b^	3,62 (0,70)
> tres SMs	3,77 (0,65)	3,46 (0,61)	3,61 (0,57)^a^	3,69 (0,54)
Valor de p	0,730	0,793	**0,005**	0,061
**Tiene acompañante en el hospital**				
Si	3,69 (0,70)	3,42 (0,66)	3,37 (0,70)	3,56 (0,70)
No	3,83 (0,60)	3,43 (0,60)	3,57 (0,78)	3,80 (0,62)
Valor de p	0,337	0,905	0,189	0,109
**Tiene comorbilidades**				
Si	3,67 (0,68)	3,44 (0,66)	3,31 (0,70)	3,48 (0,67)
No	3,76 (0,73)	3,39 (0,63)	3,55 (0,69)	3,77 (0,70)
Valor de p	0,178	0,276	**0,004**	**0,001**
**Período actual**				
Pre-operatorio	3,86 (0,64)	3,47 (0,66)	3,44 (0,77)	3,55 (0,64)
Post-operatorio	3,66 (0,70)	3,40 (0,65)	3,38 (0,69)	3,59 (0,71)
Valor de p	**0,044**	0,345	0,521	0,441

Nota: escala 6 – Capacidad para interactuar activamente con los profesionales de la salud; escala 7 – Navegar por el sistema de salud; escala 8 – Capacidad para encontrar buena información sobre salud; escala 9 – Comprender la información sobre salud y saber qué hacer; SM – salario mínimo; +Prueba U de Mann-Whitney; #Prueba de Kruskal-Wallis; datos presentados como media (desviación estándar – DE). Los valores adicionales entre paréntesis en la columna correspondiente indican el intervalo de confianza del 95% (IC95%). Las letras superpuestas (a, b, c) indican diferencias significativas entre los grupos según las comparaciones múltiples de Dunn (p < 0,05); los grupos que no comparten la misma letra difieren significativamente.

Fuente: datos de la investigación (2024).

En relación con el “cuidado activo de la salud”, la fragilidad fue más evidente en pacientes no blancos, sin comorbilidades y que se encontraban en el postoperatorio en el momento de la Recolección. Por otro lado, el “apoyo social para la salud” fue menor entre las mujeres, las personas no blancas, con ingresos de hasta un salario mínimo y los pacientes hospitalizados sin acompañante. La “capacidad para encontrar buena información sobre salud” fue menor en los participantes con menor nivel educativo y en los que utilizaban dispositivos de asistencia.

En la parte 2 del HLQ-Br, se evidenció que quienes tenían hijos obtuvieron peores puntuaciones en todas las escalas. Los datos sobre el nivel educativo mostraron que los participantes con educación primaria eran los más vulnerables a “navegar por el sistema de salud”, “encontrar buena información sobre salud” y “comprender la información sobre salud y saber qué hacer”. Los ingresos familiares de hasta tres salarios mínimos se asociaron con una mayor dificultad para encontrar buena información, y los participantes con comorbilidades mostraron una menor capacidad para encontrar y comprender la información sobre salud. Además, quienes se encontraban en el postoperatorio informaron una menor capacidad de interacción activa con los profesionales.

En la Tabla 4 se muestran las correlaciones entre el HLQ y las variables cuantitativas investigadas, como la edad, el tiempo de hospitalización y el número de personas que viven en el domicilio. Se observa una correlación positiva débil entre la edad y las escalas 1, 2, 3 y 4, una correlación negativa débil entre las escalas 5 y 8, y una correlación moderada con la escala 9. Además, se observa una correlación negativa débil entre el tiempo de hospitalización y las escalas 3, 6, 8 y 9, y también una correlación negativa débil entre el número de residentes en el domicilio y las escalas 2, 3 y 6.

**Tabla 4:** Correlación entre las escalas del *Health Literacy Questionnaire* (Cuestionario de Alfabetización en Salud), versión brasileña, y las variables cuantitativas – Santa María, RS, Brasil, 2024.

Dimensiones del HLQ-Br	Edad (años)		Tiempo de internación (días)		Número de personas viviendo en la residencia	
	r	Valor de p	r	Valor de p	r	Valor de p
**Parte 1 (escores 1–4)**						
1 – Comprensión y apoyo de los profesionales de la salud	0,207**	**< 0,001**	-0,067	0,234	-0,091	0,104
2 – Información suficiente para cuidar de la salud	0,117*	**0,037**	-0,082	0,147	-0,153*	**0,006**
3 – Cuidado activo de la salud	0,167**	**0,003**	-0,119*	**0,035**	-0,200*	**< 0,001**
4 – Apoyo social para la salud	0,144*	**0,01**	0,031	0,577	-0,005	0,927
5 – Evaluación de la información en salud	-0,124*	**0,027**	-0,1	0,076	-0,039	0,489
**Parte 2 (escores 1–5)**						
6 – Capacidad de interactuar activamente con los profesionales de salud	0,022	0,696	-0,130*	**0,021**	-0,116*	**0,038**
7 – Navegar en el sistema de salud	0,099	0,077	-0,062	0,27	-0,065	0,244
8 – Capacidad de encontrar buenas informaciones sobre salud	-0,238**	**< 0,001**	-0,152*	**0,007**	0,033	0,562
9 – Comprender las informaciones sobre salud y saber que hacer	-0,332**	**< 0,001**	-0,153*	**0,007**	0,066	0,239

r: coeficiente de correlación de Spearman *p < 0,05 y **p < 0,001.

Fuente: datos de la investigación (2024).

Tras el ajuste en la regresión lineal múltiple, la escolaridad y la edad fueron factores significativamente asociados a la escala “Comprensión y apoyo de los profesionales de la salud”. Los participantes con niveles educativos inferiores a la educación superior obtuvieron puntuaciones más bajas en esta dimensión, incluidos los analfabetos (coeficiente b = –0,87; IC del 95%: –1,41 a –0,33), los que tenían educación primaria (coeficiente b = –0,49; IC del 95%: –0,69 a –0,30) y los que tenían educación secundaria (coeficiente b = –0,28; IC del 95%: –0,48 a –0,08). Los participantes de mayor edad tienden a percibir una mejor comprensión y un mayor apoyo profesional (coeficiente b = 0,008; IC del 95%: –0,004 a 0,012).

En la escala “Información suficiente para cuidar la salud”, el nivel educativo y el número de personas en el hogar se mostraron asociados. Los participantes analfabetos (coeficiente b = –0,56; IC del 95%: –1,11 a –0,01) y con educación primaria (coeficiente b = –0,40; IC del 95%: –0,60 a –0,20) mostraron una menor percepción del acceso a la información. Además, el número de residentes en el hogar se asoció inversamente con esta percepción (coeficiente b = –0,051; IC del 95%: –0,100 a –0,001). En cuanto a la escala “Cuidado activo de la salud”, el número de personas que residen en el hogar fue el único factor asociado (coeficiente b = –0,060; IC del 95%: –0,101 a –0,018), lo que indica que residir con más personas se relacionó con un menor compromiso activo con el propio cuidado.

Para la escala “Apoyo social para la salud”, el sexo, el nivel educativo, la presencia de un acompañante y la edad fueron variables asociadas. Las mujeres presentaron un menor apoyo social (coeficiente b = –0,10; IC del 95%: –0,20 a –0,01), al igual que los participantes con educación primaria (coeficiente b = –0,29; IC del 95%: –0,47 a –0,11) y secundaria (coeficiente b = –0,31; IC del 95%: –0,49 a –0,13), en comparación con los que tenían educación superior. Contar con un acompañante se asoció con un mayor apoyo (coeficiente b = 0,22; IC del 95%: 0,06 a 0,38), y la edad mostró una relación positiva con esta dimensión (coeficiente b = 0,004; IC del 95%: 0,001 a 0,007). El nivel educativo fue el único factor asociado a la escala “Evaluación de la información en salud”, con una puntuación más baja entre los analfabetos (coeficiente b = –1,00; IC95%: –1,45 a –0,54), participantes con educación primaria (coeficiente b = –0,49; IC95%: –0,65 a –0,32) y secundaria (coeficiente b = –0,26; IC95%: –0,43 a –0,09).

En cuanto a la parte 2 del instrumento, la escala “Capacidad para interactuar activamente con los profesionales de la salud” se mostró asociada únicamente a la variable “tener hijos” (coeficiente b = –0,21; IC del 95%: –0,42 a –0,01). El nivel educativo también influyó en la escala “Navegar por el sistema de salud”, con puntuaciones más bajas entre quienes tenían educación primaria (coeficiente b = –0,29; IC del 95%: –0,53 a –0,04). Por último, en la escala “Capacidad para encontrar buena información sobre salud”, los participantes analfabetos (coeficiente b = –0,96; IC95%: –1,68 a –0,24) y con educación primaria (coeficiente b = –0,64; IC95%: –0,91 a –0,37) también obtuvieron puntuaciones más bajas. En la escala “Comprender la información sobre salud y saber qué hacer”, se observaron puntuaciones más bajas entre los analfabetos (coeficiente b = –1,29; IC del 95%: –1,97 a –0,61) y los participantes con educación primaria (coeficiente b = –0,38; IC95%: –0,63 a –0,14), además de una asociación inversa con la edad (coeficiente b = –0,004; IC95%: –0,011 a –0,001).

## DISCUSIÓN

Este estudio identificó, como potencialidades de la AS, la interacción con profesionales y el apoyo social, y como debilidades, la insuficiencia de información para el autocuidado y la dificultad para encontrar información de calidad. El análisis multivariante evidenció que el nivel educativo fue el principal factor asociado a los diferentes dominios del HLQ-Br, lo que refuerza la influencia del gradiente educativo sobre la AS. Variables como la edad, la presencia de hijos, el número de residentes en el domicilio y las comorbilidades también presentaron asociaciones relevantes, revelando matices importantes para comprender la vulnerabilidad de los pacientes quirúrgicos.

La estructura multidimensional del HLQ-Br permite una evaluación integral, que trasciende el análisis de la mera capacidad de lectura e interpretación textual, incorporando aspectos esenciales como la navegación en el sistema de salud, la interacción con los profesionales y la percepción del apoyo social. Esta característica permite identificar con precisión áreas de competencia ya consolidadas, como la comprensión adecuada de la información sanitaria, la capacidad de localizar fuentes fiables, la interacción efectiva con los profesionales, la navegación organizada por los servicios y el uso de redes de apoyo, y, simultáneamente, las debilidades que requieren la orientación de intervenciones sensibles a las particularidades socioeconómicas y culturales de los pacientes^(14)^.

El análisis de las escalas señaló como potencialidades de la AS, es decir, aspectos que se han fortalecido, el “Apoyo social para la salud” y la “Capacidad de interactuar activamente con los profesionales de la salud”, lo que indica que las redes de apoyo, tanto familiares como institucionales, pueden desempeñar un papel protector ante los retos que plantea la hospitalización. Hay pruebas sólidas que indican que las personas que se sienten apoyadas tienden a presentar una mejor adherencia terapéutica, un mayor compromiso con el autocuidado y una reducción del riesgo de complicaciones^(15,16)^, lo que reafirma el impacto positivo del apoyo social en los resultados de salud.

Las mayores limitaciones de la AS identificadas en los pacientes de este estudio fueron “Información suficiente para cuidar la salud” y “Capacidad para encontrar buena información sobre salud”, lo que indica dificultades relevantes en el acceso y uso de información para el autocuidado. Este escenario es especialmente crítico en el contexto de las cirugías digestivas, que requieren cambios complejos en el estilo de vida y una vigilancia rigurosa de las complicaciones. De este modo, esto puede ser un factor más que contribuye a aumentar la escasez de información en el periodo postoperatorio, lo que dificulta la identificación de fallos en la atención. Las medidas clínicas tradicionales no captan plenamente la experiencia del paciente, lo que limita la evaluación de la calidad centrada en la persona^(17)^.

El análisis multivariante confirmó que el nivel educativo fue la variable más fuertemente asociada con las dimensiones del HLQ-Br, incluso después de ajustar otros factores, lo que corrobora la evidencia de que el bajo nivel educativo constituye un importante predictor de menores niveles de AS^(18,19)^. Los pacientes con educación primaria obtuvieron, de manera consistente, las peores puntuaciones en varias dimensiones, seguidos por los que tenían educación secundaria, mientras que los que tenían educación superior o posgrado obtuvieron mejores resultados. Este gradiente educativo pone de manifiesto la relación entre la AS y las desigualdades sociales, en la medida en que limita el acceso, la comprensión y el uso de la información sobre salud^(20)^. Estos hallazgos están en consonancia con estudios previos, que describen que las deficiencias educativas pueden repercutir negativamente en aspectos como la autonomía y la toma de decisiones informadas, aunque estos resultados no se han evaluado directamente en este estudio^(18,19)^.

El análisis sociodemográfico añadió elementos importantes, aunque no concluyentes. Aunque la literatura suele relacionar la edad avanzada con niveles más bajos de AS, en este estudio se observó que los pacientes de más edad tendían a presentar puntuaciones más altas en dimensiones relacionadas con el apoyo social y el apoyo de los profesionales de la salud, lo que sugiere que los factores contextuales pueden atenuar o modificar este patrón. Este hallazgo es corroborado por un estudio^(21)^ que destaca el papel del apoyo social como mediador positivo de la relación entre el envejecimiento y el compromiso con el autocuidado, mitigando, en parte, las limitaciones asociadas a la edad.

Otro aspecto que merece atención se refiere a las puntuaciones medias más bajas identificadas entre los pacientes que tenían hijos, especialmente en las escalas de interacción con los profesionales, navegación en el sistema de salud y comprensión de la información, lo que sugiere una mayor vulnerabilidad en estas dimensiones de la AS. Este resultado puede estar relacionado con la sobrecarga de responsabilidades familiares, que a menudo recae sobre estas personas y puede reducir el tiempo, la atención y los recursos cognitivos dedicados a sus propias demandas de salud. Esta interpretación encuentra respaldo en la literatura, que asocia la acumulación de roles familiares y, en especial, las desigualdades de género con una menor capacidad de compromiso con las prácticas de autocuidado y la búsqueda de información en salud^(22,23)^.

Particularmente relevante fue el hallazgo de que los pacientes en el postoperatorio presentaban una fragilidad acentuada en la AS en lo que respecta al “cuidado activo de la salud” y la “capacidad de interactuar activamente con los profesionales de la salud”, hallazgo consistente con la literatura internacional^(15,16)^. Este hallazgo refuerza la necesidad de que el equipo de enfermería perioperatoria incorpore de forma sistemática la evaluación de la AS en la planificación de los cuidados, con el fin de prevenir fallos de comunicación y apoyar el cumplimiento de las recomendaciones clínicas.

La dimensión “Capacidad para encontrar buena información sobre salud”, que presentó una de las medias más bajas en el presente estudio, merece especial atención, sobre todo en el contexto contemporáneo de amplia circulación de información a menudo imprecisa, incompleta o incorrecta. Este escenario expone a los pacientes con bajo AS al riesgo de desinformación, adopción de conductas inseguras, uso inadecuado de terapias y mayor probabilidad de resultados adversos^(24,25)^.

Ante este panorama, se hace evidente la necesidad de que los servicios de salud adopten estrategias sistemáticas, continuas y estructuradas para evaluar y fortalecer la AS de los pacientes quirúrgicos. Para garantizar la seguridad, la autonomía y la adherencia a los cuidados, es fundamental adoptar prácticas como la comunicación clara, el uso de materiales educativos visuales y la validación de la comprensión mediante metodologías como el teach-back, además del apoyo educativo longitudinal^(26,27)^.

En esta lógica, con el fin de alfabetizar a los pacientes, la actuación del enfermero en el contexto del paciente quirúrgico puede beneficiarse, sobre todo, del método teach-back, una estrategia comunicativa que favorece la comprensión, la adherencia y la seguridad de la atención^(28)^. En el preoperatorio, el teach-back permite verificar si el paciente ha entendido correctamente las instrucciones sobre ayuno, higiene, uso de medicamentos y preparación emocional para la cirugía. En el postoperatorio, esta técnica ayuda a explicar los cuidados de las heridas, la administración de medicamentos, el reconocimiento de signos de complicaciones y las orientaciones para el regreso a las actividades diarias. Al pedir al paciente que repita la información con sus propias palabras, el enfermero confirma la comprensión e identifica posibles dudas, promoviendo la seguridad, la autonomía y la adherencia al tratamiento, además de contribuir a una recuperación más rápida y eficaz.

Además, los hallazgos refuerzan el papel estratégico del equipo de enfermería, que, por su proximidad con los pacientes, ocupa una posición privilegiada en la identificación de barreras para la comunicación y en el desarrollo de intervenciones educativas, colaborando directamente en la cualificación de la atención y la reducción de riesgos en el postoperatorio^(29)^.

Por último, cabe destacar que, a pesar de la creciente valoración internacional de la AS como componente central de las prácticas de atención, todavía existen lagunas en la producción de conocimiento sobre el tema en el contexto brasileño, especialmente en poblaciones quirúrgicas, lo que limita el desarrollo de políticas y prácticas basadas en la evidencia.

Este estudio presenta algunas limitaciones, una de ellas relacionada con el diseño transversal, que no permite establecer relaciones de causalidad, limitándose a la identificación de asociaciones. También se menciona la muestra por conveniencia de un único hospital universitario, lo que limita la representatividad y la generalización de los hallazgos. Aunque el HLQ-Br es un instrumento validado en Brasil, no dispone de puntos de corte establecidos que permitan categorizar el nivel de AS y comunicación, lo que limita la interpretación de los resultados a puntuaciones medias. Además, el uso de recursos visuales como apoyo para la comprensión de los ítems representó una adaptación metodológica que pudo haber influido en las respuestas. Cabe destacar también la posibilidad de sesgo de selección, por no reclutar a pacientes con limitaciones de comunicación, y de sesgo de información, ya que parte de los datos fueron autoinformados. A pesar del control de la confusión por regresión múltiple, no se puede descartar la influencia de variables no medidas.

A pesar de las limitaciones, este estudio aporta contribuciones relevantes. En la asistencia, ayuda al equipo de enfermería perioperatoria a identificar a los pacientes más vulnerables en relación con la AS y a planificar intervenciones educativas individualizadas, con un lenguaje claro, retroalimentación y materiales adaptados. En la gestión, apoya la incorporación de la evaluación de la AS como indicador de calidad y seguridad hospitalaria, orientando las políticas institucionales y los programas de capacitación. En la investigación, amplía las evidencias nacionales sobre la AS en pacientes quirúrgicos, estimulando estudios multicéntricos y longitudinales. Por último, favorece el desarrollo de prácticas personalizadas, orientadas según el perfil sociodemográfico y clínico, como estrategias diferenciadas para personas con bajo nivel educativo, refuerzo del apoyo social en pacientes sin acompañante y mayor seguimiento postoperatorio para grupos vulnerables.

## CONCLUSIÓN

El estudio identificó como potencialidades de la AS la interacción con los profesionales y el apoyo social. Como debilidades, se identificaron la insuficiencia de información para el autocuidado y la dificultad para encontrar información de calidad en el contexto de los pacientes quirúrgicos en el periodo preoperatorio o postoperatorio. El análisis multivariante evidenció que el menor nivel educativo fue el principal factor asociado a peores puntuaciones en varias dimensiones, mientras que variables como la edad, la presencia de hijos, el número de residentes en el domicilio, las comorbilidades y el periodo perioperatorio también presentaron asociaciones específicas. Estos hallazgos demuestran la influencia del gradiente educativo y las condiciones sociales sobre la AS, lo que refuerza la importancia de incorporar su evaluación sistemática a la práctica de la enfermería perioperatoria y de desarrollar intervenciones educativas accesibles y centradas en el paciente.

## DISPONIBILIDAD DE DATOS

El conjunto de datos que respaldan las conclusiones de este estudio está disponible previa solicitud al autor correspondiente.
